# A positive feed-forward loop between *Fusobacteriumnucleatum* and ethanol metabolism reprogramming drives laryngeal cancer progression and metastasis

**DOI:** 10.1016/j.isci.2022.103829

**Published:** 2022-01-30

**Authors:** Chi-Yao Hsueh, Qiang Huang, Hongli Gong, Yujie Shen, Ji Sun, Hui-Ching Lau, Duo Zhang, Di Tang, Chunping Wu, Yang Guo, Huiying Huang, Pengyu Cao, Lei Tao, Ming Zhang, Liang Zhou

**Affiliations:** 1 Department of Otorhinolaryngology, Eye & ENT Hospital, Fudan University, 83 Fen Yang Road, Shanghai 200031, China; 2 Department of Pathology, Eye & ENT Hospital, Fudan University, 83 Fen Yang Road, Shanghai 200031, China

**Keywords:** Microbial metabolism, Mycology, Cancer

## Abstract

Alcohol consumption, which affects the structure and composition of the laryngeal microbiota, is one of the most important risk factors for laryngeal squamous cell cancer (LSCC). Our results demonstrated that high enrichment of *Fusobacterium nucleatum* (*F. nucleatum*) in LSCC was associated with poor prognosis. *F. nucleatum* increased miR-155-5p and miR-205-5p expression to suppress alcohol dehydrogenase 1B (ADH1B) and transforming growth factor β receptor 2 (TGFBR2) expression by activating innate immune signaling, resulting in ethanol metabolism reprogramming to allow *F. nucleatum* accumulation and PI3K/AKT signaling pathway activation to promote epithelial-mesenchymal transition, further exacerbating the uncontrolled progression and metastasis of LSCC. Therefore, the positive feed-forward loop between *F. nucleatum* and ethanol metabolism reprogramming promotes cell proliferation, migration, and invasion to affect LSCC patient prognosis. The amount of *F. nucleatum* is a potential prognostic biomarker, which yields valuable insight into clinical management that may improve the oncologic outcome of patients with LSCC.

## Highlights

• Identification of *Fusobacterium nucleatum* as a risk factor for recurrence in LSCC patients
• The positive feed-forward loop between *F. nucleatum* and ethanol metabolism reprogramming
• *F. nucleatum* suppresses TGFBR2, resulting in EMT in LSCC

## Graphical Abstract

**Figure:**
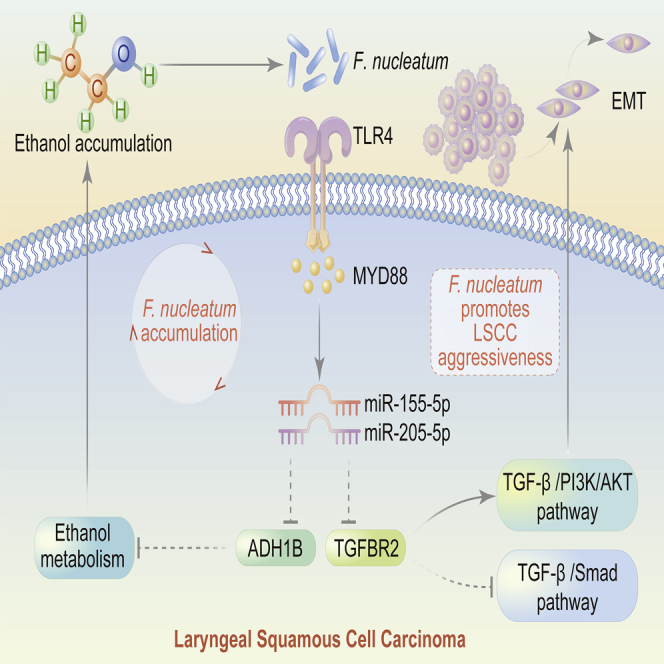

## Introduction

Laryngeal cancer is the second most common malignant tumor of the respiratory system in the United States, with an estimated 12,620 new cases and 3,770 cancer-related deaths in 2021 (Siegel et al., 2021). The most common pathological type (>95%) of laryngeal cancer is laryngeal squamous cell cancer (LSCC) (Marioni et al., 2006). Unfortunately, LSCC remains one of few cancer types with no improvement in the 5-year survival rate over the past 40 years (Siegel et al., 2016). The currently accepted treatments, including surgery, radiotherapy, and chemotherapy either alone or in combination, have improved survival and life expectancy, but patients with LSCC with advanced-stage disease still do not have an optimistic prognosis and experience loss of normal laryngeal function (Steuer et al., 2017). Thus, it is of paramount importance to elucidate the molecular mechanism underlying LSCC to develop therapeutic strategies that can halt or slow disease progression and retain normal physiological laryngeal function.

The laryngeal cavity is a region of the upper respiratory tract that harbors various microbial populations (Li et al., 2012). Poor lifestyle habits, especially alcohol consumption, have been found to affect the structure and composition of the oral microbiota (Fan et al., 2018; Hsiao et al., 2018). Our previous study demonstrated that *Fusobacterium* abundance was higher in LSCC tissues than in para-cancerous normal epithelial tissues and vocal cord polyp tissues (Gong et al., 2013, 2014, 2017; Hsueh et al., 2020). *Fusobacterium* *nucleatum* (*F. nucleatum*) is one of the most important species of *Fusobacterium* and is generally considered a factor strongly related to tumor progression and treatment response in alimentary and respiratory cancers (Brennan and Garrett, 2019; Han, 2015). Several lines of evidence suggest that *F. nucleatum* plays a vital role in the regulation of miRNAs to promote tumor proliferation and invasive activities and increases the risk of recurrence and chemoresistance by activating the myeloid differentiation 88 (MYD88)-dependent Toll-like receptor 4 (TLR4)-mediated innate immune signaling pathway (Yang et al., 2017; Yu et al., 2017). Aggressive LSCC phenotypes rely mainly on epithelial-mesenchymal transition (EMT) (Pastushenko and Blanpain, 2019), and *F. nucleatum*, as a component of the tumor microenvironment, may influence the prognosis of patients with LSCC by regulating genes related to EMT. However, the molecular mechanism by which *F. nucleatum* promotes EMT in LSCC has not been established.

In this study, we attempt to clarify why *F. nucleatum* accumulates in LSCC and how it influences LSCC aggressiveness. We also aimed to decipher the mechanism of the positive feed-forward loop between *F. nucleatum* and ethanol metabolism reprogramming that promotes EMT in LSCC. Our results show that *F. nucleatum* shows considerable potential as a prognostic biomarker and therapeutic target in patients with LSCC.

## Results

### A high *F. nucleatum* amount is associated with high alcohol consumption and recurrence in patients with LSCC

**Figure 1:**
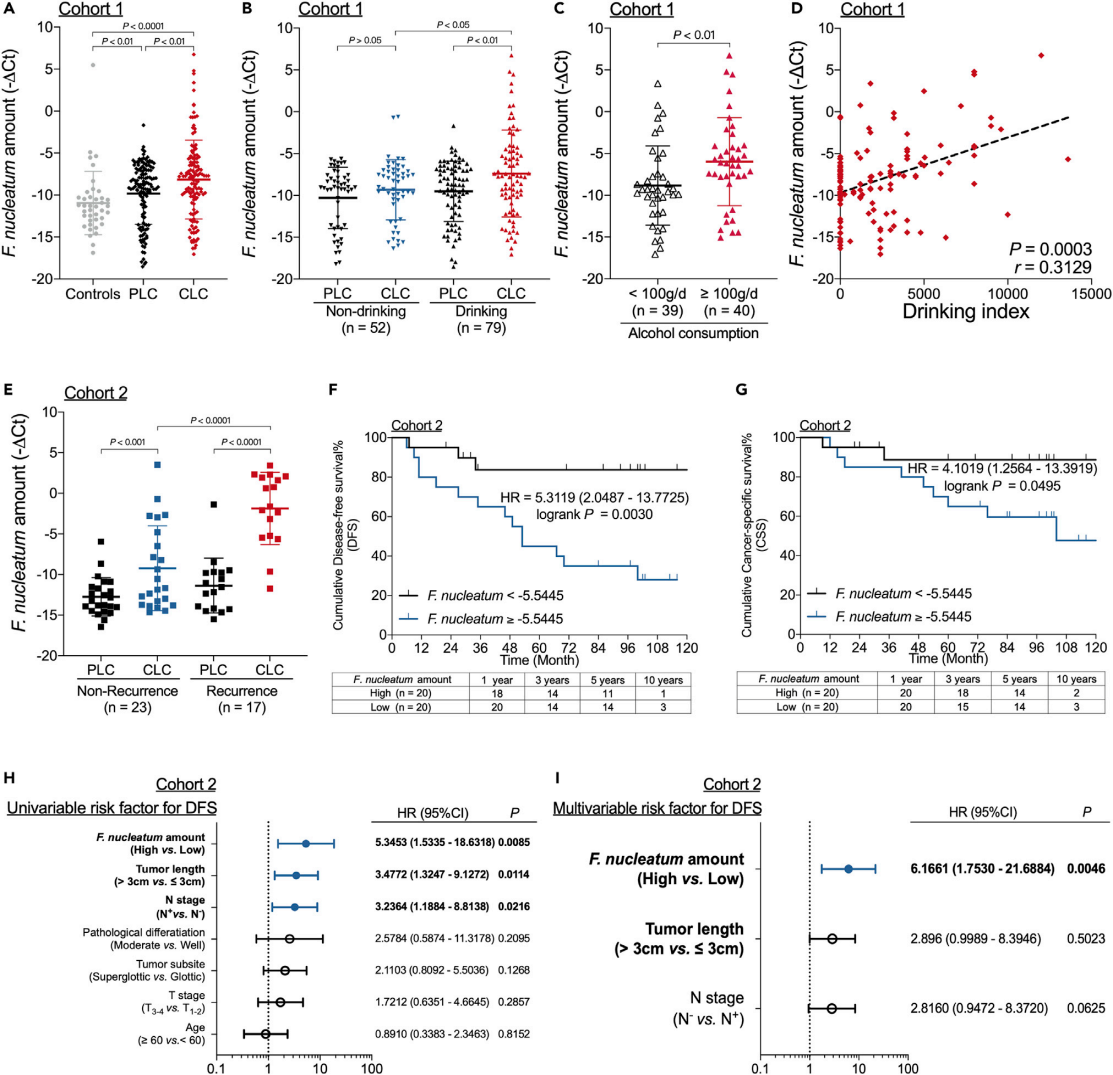
A high *F. nucleatum* amount is associated with high alcohol consumption and recurrence in patients with LSCC (A) Statistical analysis of the amount of *F. nucleatum* in samples from Cohort 1 as calculated with the Wilcoxon matched pairs signed-rank test and Mann-Whitney test. (B) Statistical analysis of the association of the amount of *F. nucleatum* in patients with LSCC with history of alcohol consumption as calculated with the Wilcoxon matched pairs signed-rank test and Mann-Whitney test. (C) Statistical analysis of the association of the amount of *F. nucleatum* in LSCC tissues with alcohol consumption (grams per day) in patients from Cohort 1 as calculated with the Mann-Whitney test. (D) The correlation between the amount of *F. nucleatum* in LSCC tissues and the drinking index (average alcohol in grams per day multiplied by years of drinking) in Cohort 1 as assessed with the two-tailed nonparametric Spearman correlation coefficient. (E) Statistical analysis of the *F. nucleatum* content and recurrence in patients with LSCC from Cohort 2 as calculated with the Wilcoxon matched pairs signed-rank test and Mann-Whitney test. (F) Disease-free survival (DFS) of patients in Cohort 2 with high and low amounts of *F. nucleatum* as assessed by the log rank test. (G) Cancer-specific survival (CSS) of patients in Cohort 2 with high and low amounts of *F. nucleatum* as assessed by the log rank test. (H) Univariate analysis for DFS was performed on patients from Cohort 2. The bars correspond to 95% confidence intervals. (I) Multivariate analysis for DFS was performed on patients from Cohort 2. The bars correspond to 95% confidence intervals. Data are represented as mean ± SD.

The amount of *F. nucleatum* was higher in LSCC tissues (CLC) than in paired para-cancerous normal epithelial tissues (PLC) and fresh vocal cord polyp tissues (Controls) in Cohort 1 (Figure 1A). In the CLC group from Cohort 1, the amount of *F. nucleatum* was higher in patients with a history of alcohol consumption than in those without a history of alcohol consumption (Figure 1B and Table S1), and patients with LSCC with heavy alcohol consumption (≥100 g per day) had more *F. nucleatum* than those without heavy alcohol consumption (<100 g per day) (Figure 1C). Furthermore, the amount of *F. nucleatum* in LSCC tissues was positively correlated with the drinking index (Figure 1D). Multiple linear regression models with the *F. nucleatum* amount as the dependent variable showed a significant correlation between the drinking index and *F. nucleatum* amount in patients with LSCC (Table S2), indicating a correlation between *F. nucleatum* content and alcohol consumption.

We next investigated the correlation between the amount of *F. nucleatum* and prognosis in patients with LSCC using a different cohort (Cohort 2). The amount of *F. nucleatum* in CLC was higher in patients with recurrence than in patients without recurrence (Figure 1E and Table S3). When patients with LSCC were stratified according to the median *F. nucleatum* amount, those in the high *F. nucleatum* group had substantially shorter disease-free survival (DFS, Figure 1F) and cancer-specific survival (CSS, Figure 1G) than those in the low *F. nucleatum* group. Furthermore, the amount of *F. nucleatum* was identified as an independent predictor for DFS by univariate and multivariate Cox proportional hazards (CPH) regression analyses of Cohort 2 (Figures 1H and 1I).

### The positive feed-forward loop between *F. nucleatum* and ethanol metabolism reprogramming promotes LSCC aggressiveness

**Figure 2:**
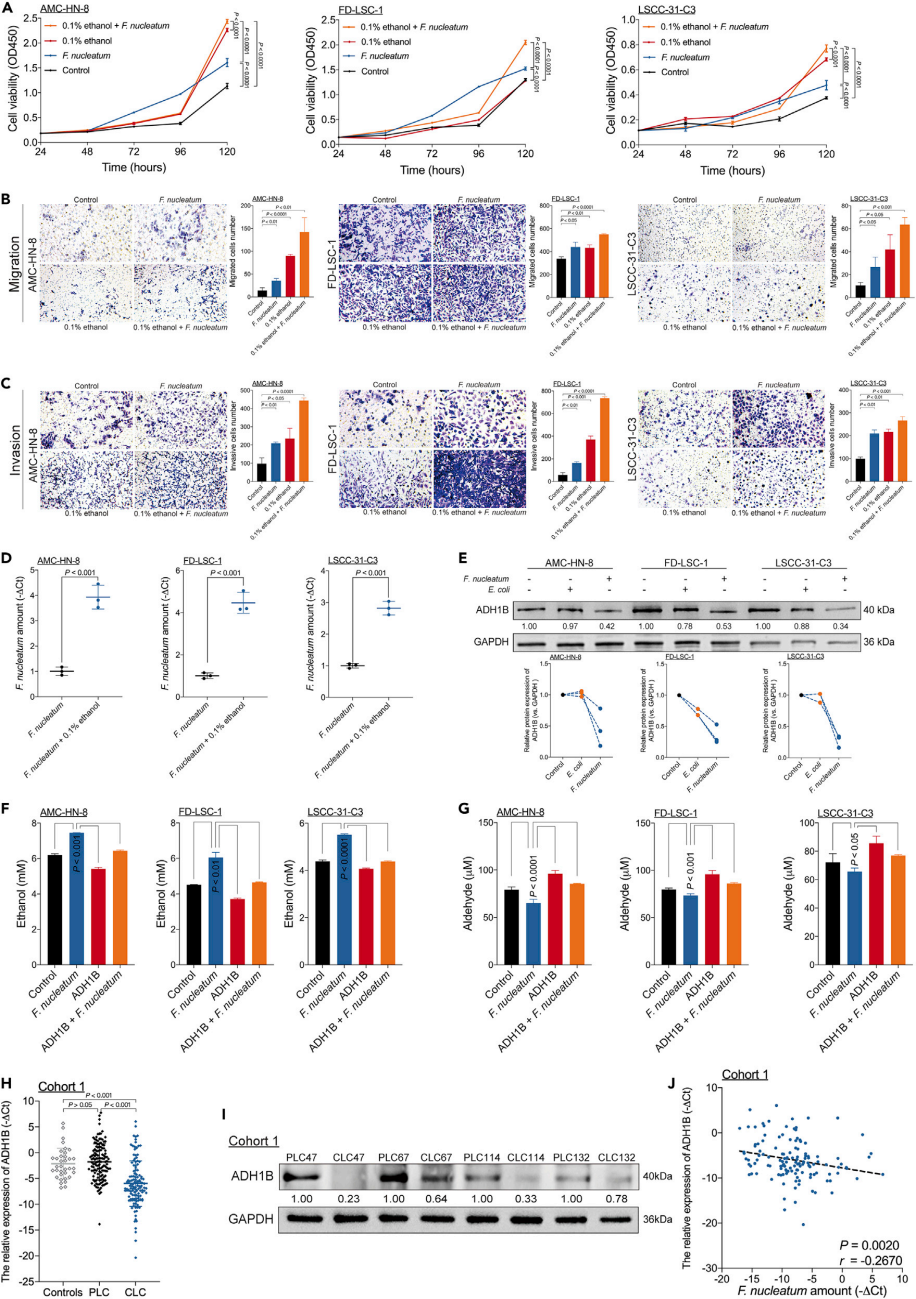
The positive feed-forward loop between *F. nucleatum* and ethanol metabolism reprogramming promotes LSCC aggressiveness (A) A CCK-8 assay was performed in three LSCC cell lines cultured in the presence or absence of *F. nucleatum* and 0.1% (v/v) ethanol as assessed by the ordinary two-way ANOVA. (B) Transwell migration assays were performed in three LSCC cell lines cultured in the presence or absence of *F. nucleatum* and 0.1% (v/v) ethanol as calculated with the Student’s unpaired t test. (C) Transwell invasion assays were performed in three LSCC cell lines cultured in the presence or absence of *F. nucleatum* and 0.1% (v/v) ethanol as calculated with the Student’s unpaired t test. (D) Statistical analysis of the amount of *F. nucleatum* in three LSCC cell lines cocultured with *F. nucleatum* and treated with 0.1% (v/v) ethanol as calculated with Student’s unpaired t test. (E) Western blotting was performed to assess ADH1B expression in three LSCC cell lines cocultured with *F. nucleatum* or *E. coli*. (F) The ethanol content was measured by colorimetric assay in three LSCC cells with or without ADH1B-overexpressing lentiviral vector transduction and subsequently cocultured with *F. nucleatum* and 0.1% (v/v) ethanol as assessed by the ordinary one-way ANOVA. (G) The aldehyde content was measured by colorimetric assay in three LSCC cell lines with or without transduced ADH1B-overexpressing lentiviral vector and subsequent coculture with *F. nucleatum* and 0.1% (v/v) ethanol as assessed by the ordinary one-way ANOVA. (H) Statistical analysis of ADH1B expression in samples from Cohort 1 as calculated with the Wilcoxon matched pairs signed-rank test and Mann-Whitney test. (I) Western blotting for ADH1B expression was performed on samples from Cohort 1. (J) The correlation between the amount of *F. nucleatum* and ADH1B expression in LSCC tissues of Cohort 1 as assessed by the two-tailed nonparametric Spearman correlation coefficient. Data are represented as mean ± SD.

Based on the positive correlation between the *F. nucleatum* content and alcohol consumption, we hypothesized that ethanol promoted *F. nucleatum* colony formation, and we observed this exact scenario (Figure S1). We next posited that *F. nucleatum* and ethanol were biologically involved in the development of LSCC. To test this hypothesis, we cocultured LSCC cells in the presence or absence of *F. nucleatum* and/or 0.1% (v/v) ethanol to evaluate the cells' proliferation, migration, and invasion abilities. *F. nucleatum* and 0.1% (v/v) ethanol individually promoted cell proliferation (Figure 2A), migration (Figure 2B), and invasion (Figure 2C), whereas the combination of *F. nucleatum* and 0.1% (v/v) ethanol further enhanced LSCC aggressiveness (Figures 2A–2C). However, coculture of LSCC cells with *Escherichia coli* did not promote LSCC cell migration and invasion (Figure S2). During a 3-day incubation period, three LSCC cell lines cocultured with 0.1% ethanol promoted *F. nucleatum* proliferation in an aerobic environment (Figure 2D). Thus, ethanol and ethanol-mediated *F. nucleatum* accumulation cooperatively promote LSCC aggressiveness.

Next, we investigated whether *F. nucleatum* can modulate ethanol metabolism in LSCC cells. Seven genes of the alcohol dehydrogenase (ADH) family, along with cytochrome P450 family 2 subfamily E member 1 (CYP2E1), are responsible for the majority of ethanol oxidation to acetaldehyde; we observed that only the alcohol dehydrogenase 1B (ADH1B) gene was consistently downregulated in LSCC cells cocultured with *F. nucleatum* (Figure S3A). LSCC cells cocultured with *F. nucleatum* exhibited decreased ADH1B expression (Figures 2E, S3B, and S4). Moreover, LSCC coculture with *F. nucleatum* resulted in decreased enzymatic activity of ADH family members (Figure S3C). Consistent with this, coculture of LSCC cells with *F. nucleatum* increased the ethanol content (Figure 2F) but decreased the aldehyde content (Figure 2G). These data indicate the existence of a positive feed-forward loop between *F. nucleatum* and ethanol metabolism reprogramming in LSCC cells.

We next evaluated the relationship between *F. nucleatum* content and ADH1B expression in patients with LSCC. ADH1B expression was decreased in CLC compared with PLC and control samples from Cohort 1 (Figure 2H), with the protein and RNA expression levels of ADH1B showing general consistency (Figure 2I). Moreover, the amount of *F. nucleatum* was negatively correlated with ADH1B expression (Figure 2J).

### *F. nucleatum* induces switching from the Smad-dependent to the Smad-independent TGF-β signaling to promote EMT in LSCC by suppressing TGFBR2 expression

**Figure 3:**
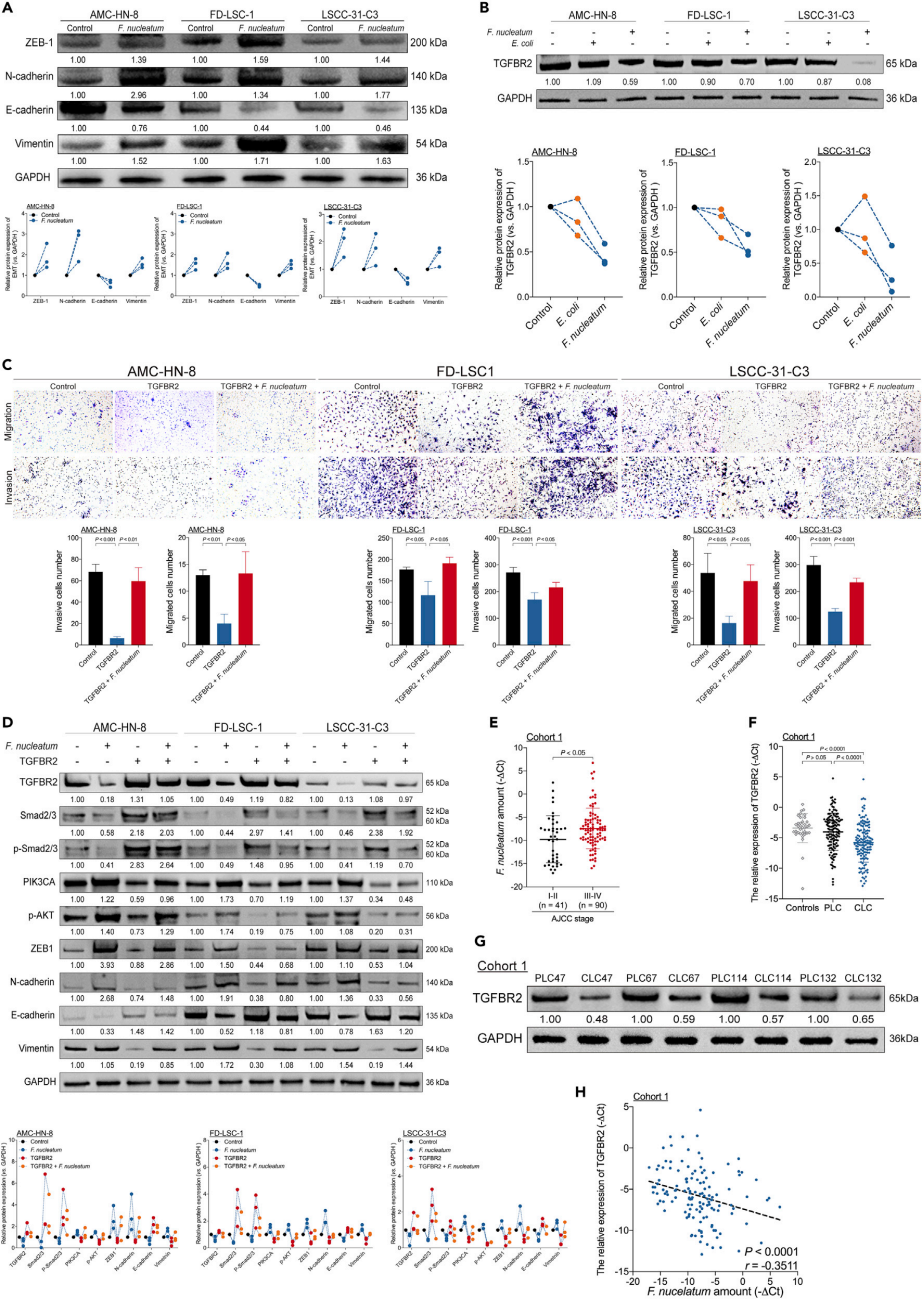
*F. nucleatum* induces switching from the Smad-dependent to the Smad-independent TGF-β signaling to promote EMT in LSCC by suppressing TGFBR2 expression (A) Western blotting was performed to evaluate E-cadherin, N-cadherin, Vimentin, and ZEB-1 expression in three LSCC cell lines cocultured with *F. nucleatum*. (B) Western blotting was performed to assess TGFBR2 expression in three LSCC cell lines cocultured with *F. nucleatum* or *E. coli*. (C) Transwell migration and invasion assays were performed in three LSCC cell lines with or without transduced TGFBR2-overexpressing lentiviral vector and subsequent coculture with *F. nucleatum* as calculated with the Student’s unpaired t test. (D) Western blotting was performed on Smad-dependent TGF-B signaling-related proteins (Smad2/3 and p-Smad2/3), PI3K/AKT signaling-related proteins (PI3KCA and p-AKT), and EMT-related proteins (E-cadherin, N-cadherin, Vimentin, and ZEB-1) in three LSCC cell lines with or without transduced TGFBR2-overexpressing lentiviral vector and subsequent coculture with *F. nucleatum*. (E) Statistical analysis of the amount of *F. nucleatum* stratified by TNM tumor stage in LSCC tissues from Cohort 1 as calculated with the Mann-Whitney test. (F) Statistical analysis of TGFBR2 expression in tissue samples from Cohort 1 as calculated with the Wilcoxon matched pairs signed-rank test and Mann-Whitney test. (G) Western blotting for TGFBR2 expression was performed on samples from Cohort 1. (H) The correlation between the amount of *F. nucleatum* and TGFBR2 expression in LSCC tissues of Cohort 1 as assessed by the two-tailed nonparametric Spearman correlation coefficient. Data are represented as mean ± SD.

EMT is a mechanism underlying tumor migration and invasion in LSCC (Pastushenko and Blanpain, 2019). Consistent with this observation, coculture of LSCC cells with *F. nucleatum* affected the expression of EMT-related molecules. *F. nucleatum* increased N-cadherin, Vimentin, and ZEB-1 expression but decreased E-cadherin expression (Figures 3A, S5A, and S5B) in LSCC cells. The expression of transforming growth factor β receptor 2 (TGFBR2), a tumor suppressor, is downregulated in head and neck squamous cell cancer (HNSCC) (Chu et al., 2013) and is generally related to EMT in LSCC (Lu et al., 2006). In line with these results, we show that coculture of LSCC cells with *F. nucleatum* inhibited TGFBR2 expression (Figures 3B, S4, S6A, and S6B), whereas TGFBR2 overexpression inhibited *F. nucleatum*-induced migration and invasion of LSCC cells (Figure 3C). Moreover, TGFBR2 overexpression decreased N-cadherin, Vimentin, and ZEB-1 expression but increased E-cadherin expression in three LSCC cell lines (Figure S6C). Thus, *F.* nucleatum induces EMT and promotes cell migration and invasion in LSCC by suppressing TGFBR2 expression.

The PI3K/AKT signaling pathway is a central regulator of HNSCC progression (Wang et al., 2021). Multiple studies have suggested the existence of direct or indirect cross talk between TGF-β signaling and PI3K/AKT signaling (Gu and Feng, 2018; Xie et al., 2018). Thus, we speculated that LSCC aggressiveness is promoted via the PI3K/AKT signaling pathway in the presence of *F. nucleatum*-mediated inactivation of TGFBR2. *F. nucleatum* increased the protein levels of PIK3CA, p-AKT, N-cadherin, Vimentin, and ZEB-1 but decreased the protein levels of Smad2/3, p-Smad2/3, and E-cadherin. Conversely, TGFBR2 overexpression decreased the protein levels of PIK3CA, p-AKT, N-cadherin, Vimentin, and ZEB-1 but increased the protein levels of Smad2/3, p-Smad2/3, and E-cadherin (Figure 3D). Thus, *F. nucleatum* induces switching from Smad-dependent to Smad-independent TGF-β signaling to promote EMT in LSCC by suppressing TGFBR2 expression.

We next evaluated the relationship between the *F. nucleatum* amount, tumor-node-metastasis (TNM) stage, and TGFBR2 expression level in patients with LSCC from Cohort 1. The amount of *F. nucleatum* was higher in advanced-stage LSCC than in early-stage LSCC samples (Figure 3E). TGFBR2 expression was decreased in LSCC compared with PLC and control samples (Figure 3F). The protein expression level of TGFBR2 was generally consistent with its RNA expression level (Figure 3G). Moreover, the amount of *F. nucleatum* in CLC tissues was negatively correlated with the expression level of TGFBR2 (Figure 3H).

### *F. nucleatum* inhibits ADH1B and TGFBR2 expression via selective gain of miR-155-5p and miR-205-5p via the TLR4/MYD88 signaling pathway

We explored the mechanism by which *F. nucleatum* mediates ADH1B and TGFBR2 expression. Recent research has indicated that *F. nucleatum* promotes cancer proliferation and chemoresistance by regulating multiple miRNAs (Yang et al., 2017; Yu et al., 2017). The results of the high-throughput screen in our previous study showed that *F. nucleatum* increased miR-155-5p and miR-205-5p expression (Figure S7A) (Cao et al., 2013). Moreover, miR-155-5p and miR-205-5p inhibitors suppressed *F. nucleatum*-mediated expression of miR-155-5p and miR-205-5p (Figure S7B).

**Figure 4:**
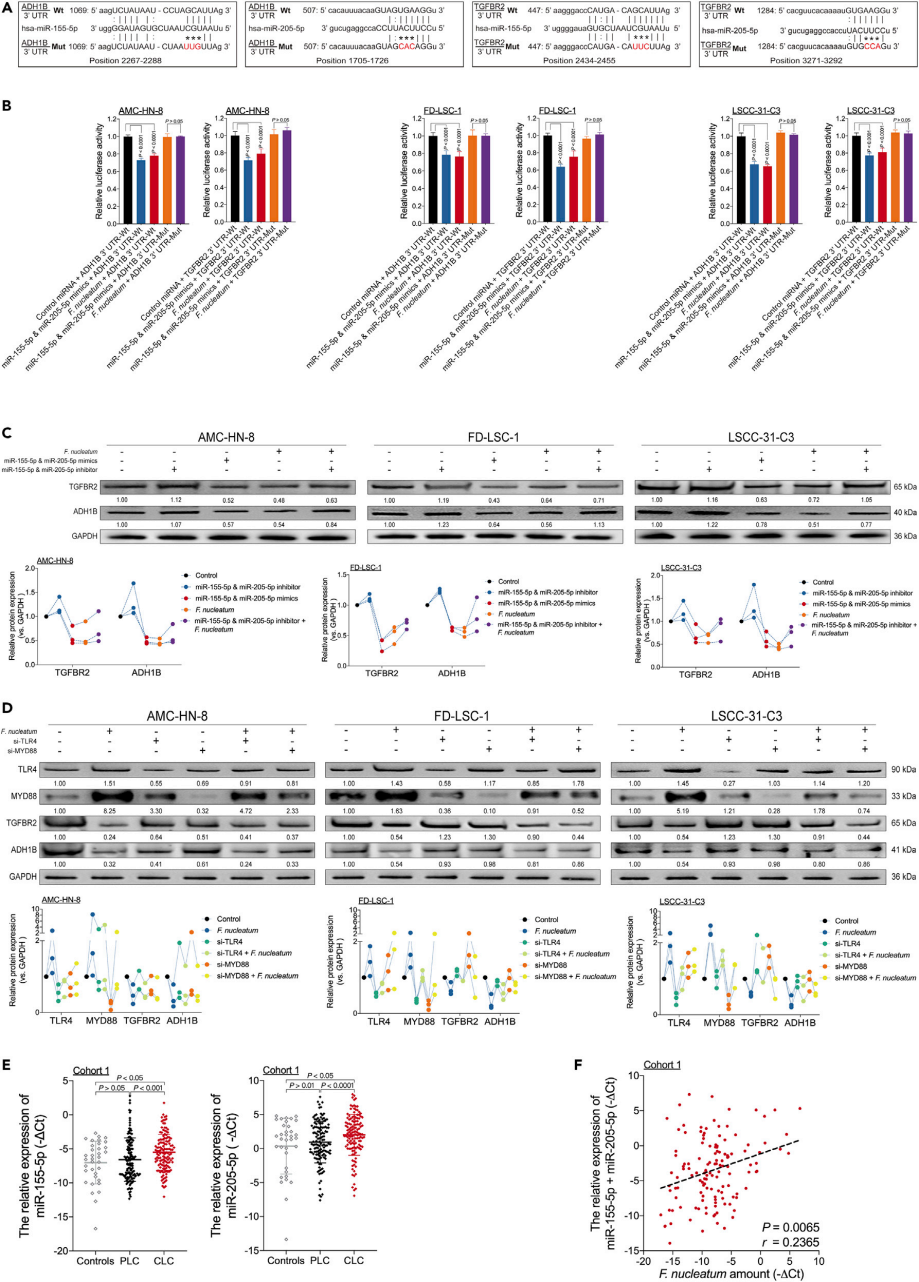
*F. nucleatum* inhibits ADH1B and TGFBR2 expression via selective gain of miR-155-5p and miR-205-5p via the TLR4/MYD88 signaling pathway (A) The predicted binding sequences for miR-155-5p and miR-205-5p within the 3′ UTRs of human ADH1B and TGFBR2 mRNA. Seed sequences are highlighted. (B) Dual-luciferase activity was measured in three LSCC cell lines cocultured with *F. nucleatum* or treated with miR-155-5p and miR-205-5p mimics or control miRNA. Dual-luciferase reporters expressing wild-type (Wt) or mutant (Mut) 3′ UTRs from human ADH1B and TGFBR2 mRNA were used. The dual-luciferase activity was normalized based on the control miRNA transfection as assessed by the ordinary one-way ANOVA. (C) Western blotting was performed for ADH1B and TGFBR2 in three LSCC cell lines transfected with miR-155-5p and miR-205-5p mimics or inhibitor and cultured in the presence or absence of *F. nucleatum*. (D) Western blotting for ADH1B and TGFBR2 expression was performed in three LSCC cell lines transfected with TLR4 and MYD88 siRNAs and cultured in the presence or absence of *F. nucleatum*. (E) Statistical analysis of the relative expression of miR-155-5p (left) and miR-205-5p (right) in samples from Cohort 1 as calculated with the Wilcoxon matched pairs signed-rank test and Mann-Whitney test. (F) Correlation between the amount of *F. nucleatum* and the relative expression of miR-155-5p and miR-205-5p in LSCC tissues from Cohort 1 as assessed by the two-tailed nonparametric Spearman correlation coefficient. Data are represented as mean ± SD.

The target prediction program mirDIP and a sorting algorithm were used to predict potential specific binding sites for miR-155-5p and miR-205-5p in the seed regions within the 3′ UTRs of ADH1B and TGFBR2 mRNA (Tokar et al., 2018) (Figure 4A). The dual-luciferase reporter assay results demonstrated that high levels of *F. nucleatum*, miR-155-5p, and miR-205-5p suppressed luciferase activity in HEK293T (Figure S7C) and three LSCC cells (Figure 4B) transfected with the wild-type ADH1B or TGFBR2 reporter plasmids but not with the corresponding mutant reporter plasmids. Moreover, *F. nucleatum*, miR-155-5p, and miR-205-5p decreased ADH1B and TGFBR2 expression in LSCC cells, which was rescued by treatment with miR-155-5p and miR-205-5p inhibitors (Figures 4C and S7D). Thus, these data indicate that *F. nucleatum* inhibits ADH1B and TGFBR2 expression by increasing miR-155-5p and miR-205-5p expression.

Since coculture of LSCC cells with *F. nucleatum* promotes metabolic reprogramming and EMT in LSCC by upregulating miR-155-5p and miR-205-5p, we speculated that the TLR4/MYD88 pathway is activated by *F. nucleatum*. Consistent with our hypothesis, *F. nucleatum* increased TLR4 and MYD88 expression in cocultured LSCC cells (Figures S8A and S8B). By contrast, LSCC cells transfected with siRNA targeting TLR4 or MYD88 exhibited decreased miR-155-5p and miR-205-5p expression, which was rescued when the cells were cocultured with *F. nucleatum* (Figure S8C). Moreover, LSCC cells transfected with siRNA against TLR4 or MYD88 exhibited increased ADH1B and TGFBR2 expression, which was rescued when the cells were cocultured with *F. nucleatum* (Figures 4D and S8D). Thus, these data indicate that *F. nucleatum*-induced expression of miR-155-5p and miR-205-5p results in ethanol metabolism reprogramming in LSCC via inhibition of ADH1B expression and in EMT via inhibition of TGFBR2 expression via the TLR4/MYD88 signaling pathway.

We next evaluated the relationship between the *F. nucleatum* content and the expression levels of miR-155-5p and miR-205-5p in patients with LSCC from Cohort 1. The miR-155-5p and miR-205-5p expression levels were increased in CLC samples compared with PLC and control samples (Figure 4E). Moreover, the amount of *F. nucleatum* in the CLC samples was positively correlated with the combined expression level of miR-155-5p and miR-205-5p (Figure 4F).

### *F. nucleatum* promotes LSCC tumor proliferation and metastasis by suppressing TGFBR2 expression

To investigate the capacity of *F. nucleatum* to promote tumor proliferation and metastasis, we established subcutaneous xenograft and metastasis models of LSCC cells cocultured with *F. nucleatum* in BALB/c nude mice.

**Figure 5:**
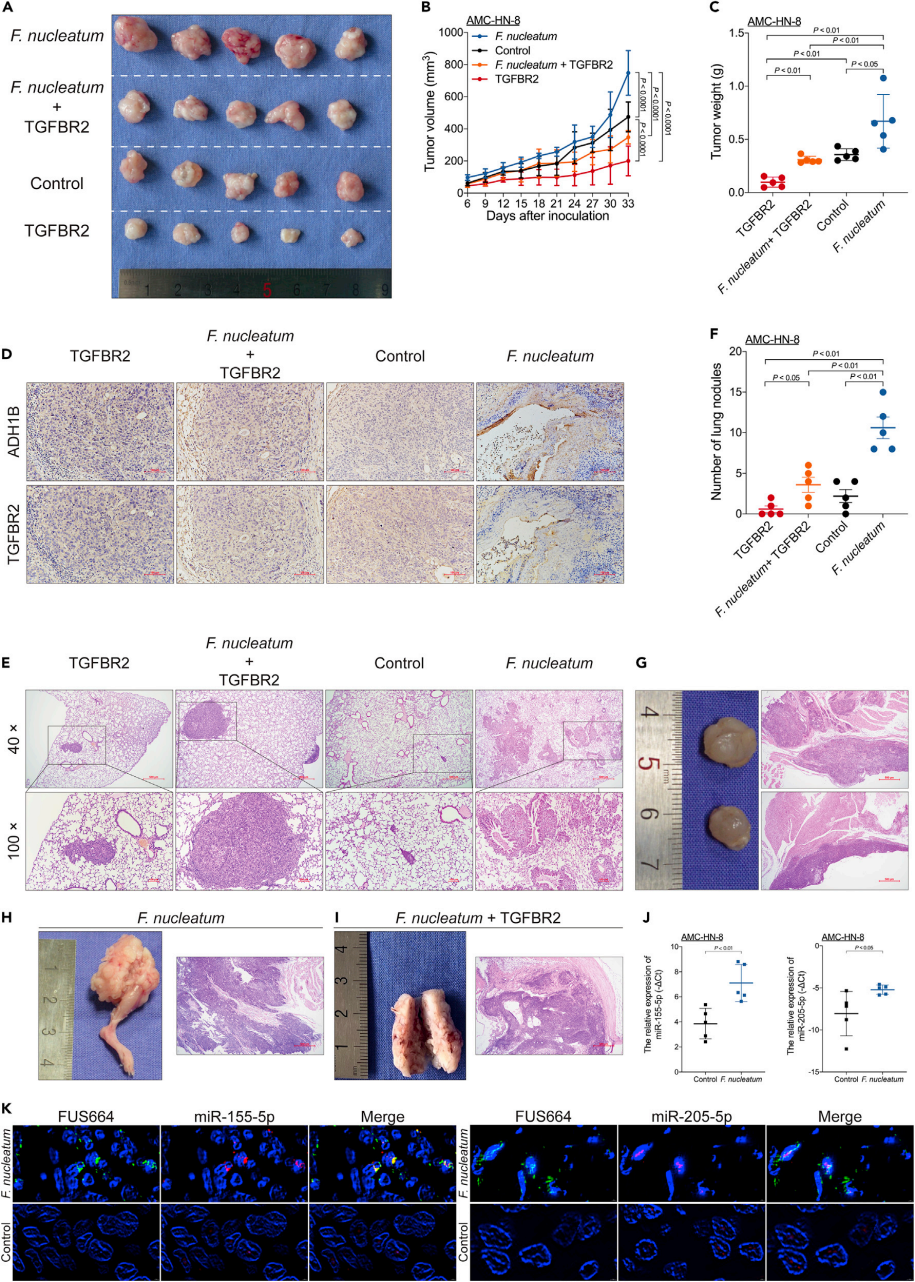
*F. nucleatum* promotes LSCC tumor proliferation and metastasis by suppressing TGFBR2 expression (A) Representative data of tumors derived from AMC-HN-8 cells subjected to different experimental conditions (n = 5 for each mouse group). (B) Statistical analysis of tumor volumes from mice subjected to different experimental conditions (n = 5 for each group) as calculated with the ordinary two-way ANOVA. (C) Statistical analysis of tumor weights from mice subjected to different experimental conditions (n = 5 for each group) as calculated with the Mann-Whitney test. (D) Representative immunohistochemical staining (10 × magnification) for ADH1B and TGFBR2 expression in tumors from mice subjected to different experimental conditions. Scale bar, 100 μm. (E) Representative data of hematoxylin-eosin (H&E)-stained sections of metastatic nodules from lungs. (F) Statistical analysis of the number of metastatic nodules in lungs from mice subjected to different experimental conditions (n = 5 for each group) as calculated with the Mann-Whitney test. (G) Representative data of metastatic nodules in the neck (n = 2, left) and H&E-stained sections (40 × magnification, right) from mice in the *F. nucleatum* group. Scale bar, 500 μm. (H) Representative data of metastatic nodules in the right thigh (n = 1, left) and H&E-stained sections (40 × magnification, right) from mice in the *F. nucleatum* group. Scale bar, 500 μm. (I) Representative data of metastatic nodules in the right thigh (n = 1, left) and H&E-stained sections (40 × magnification, right) from mice in the *F. nucleatum* + TGFBR2 group. Scale bar, 500 μm. (J) Relative expression of miR-155-5p (left) and miR-205-5p (right) in tumors from mice subjected to different experimental conditions (n = 5 for each group) as calculated with the Mann-Whitney test. (K) Representative fluorescence *in situ* hybridization (FISH) assays are shown (400 × magnification) for miR-155-5p (Cy3-labeled, left) and miR-205-5p (Cy3-labeled, right) expression in LSCC cells cultured in the presence or absence of *F. nucleatum* (FUS664, FAM-labeled). Scale bar, 2 μm. Data are represented as mean ± SD.

In the xenograft mouse model, AMC-HN-8 cells with or without the transduced lentiviral TGFBR2 overexpression vector and cultured in the presence or absence of *F. nucleatum* were inoculated into nude mice. The xenografts in mice inoculated with AMC-HN-8 cells cocultured with *F. nucleatum* had larger volumes and weights than those in mice from the other three groups. In addition, *F. nucleatum*-induced LSCC cell proliferation was reversed by TGFBR2 overexpression (Figures 5A–5C). Immunohistochemical (IHC) staining also showed that *F. nucleatum*-induced LSCC cell proliferation was abolished by TGFBR2 overexpression (Figure 5D).

In the metastasis experiment, the number of lung metastatic nodules was higher in mice inoculated with AMC-HN-8 cells cocultured with *F. nucleatum* than in mice from the other three groups. In addition, *F. nucleatum*-induced lung metastasis was reversed by TGFBR2 overexpression (Figures 5E–5F). Interestingly, two nude mice in the *F. nucleatum* group had concurrent metastases, one in the lung and neck and the other in the lung and right thigh (Figures 5G and 5H). In addition, one nude mouse in the *F. nucleatum* + TGFBR2 group had concurrent metastasis in the lung and right thigh (Figure 5I). These data indicate that *F. nucleatum* promotes LSCC proliferation and metastasis via selective loss of TGFBR2 expression.

We further investigated whether *F. nucleatum* promotes miR-155-5p and miR-205-5p expression in the subcutaneous xenograft model of LSCC. Indeed, LSCC cells cocultured with *F. nucleatum* exhibited increased miR-155-5p and miR-205-5p expression (Figure 5J). Moreover, fluorescence *in situ hybridization* (FISH) revealed that *F. nucleatum* promoted miR-155-5p and miR-205-5p expression (Figure 5K). Taken together, these data indicate that miR-155-5p and miR-205-5p regulate *F. nucleatum*-mediated LSCC proliferation and metastasis by suppressing TGFBR2 expression.

### The *F. nucleatum* content correlates with the miR-155-5p, miR-205-5p, ADH1B, and TGFBR2 levels in LSCC and indicates poor oncologic outcome

**Figure 6:**
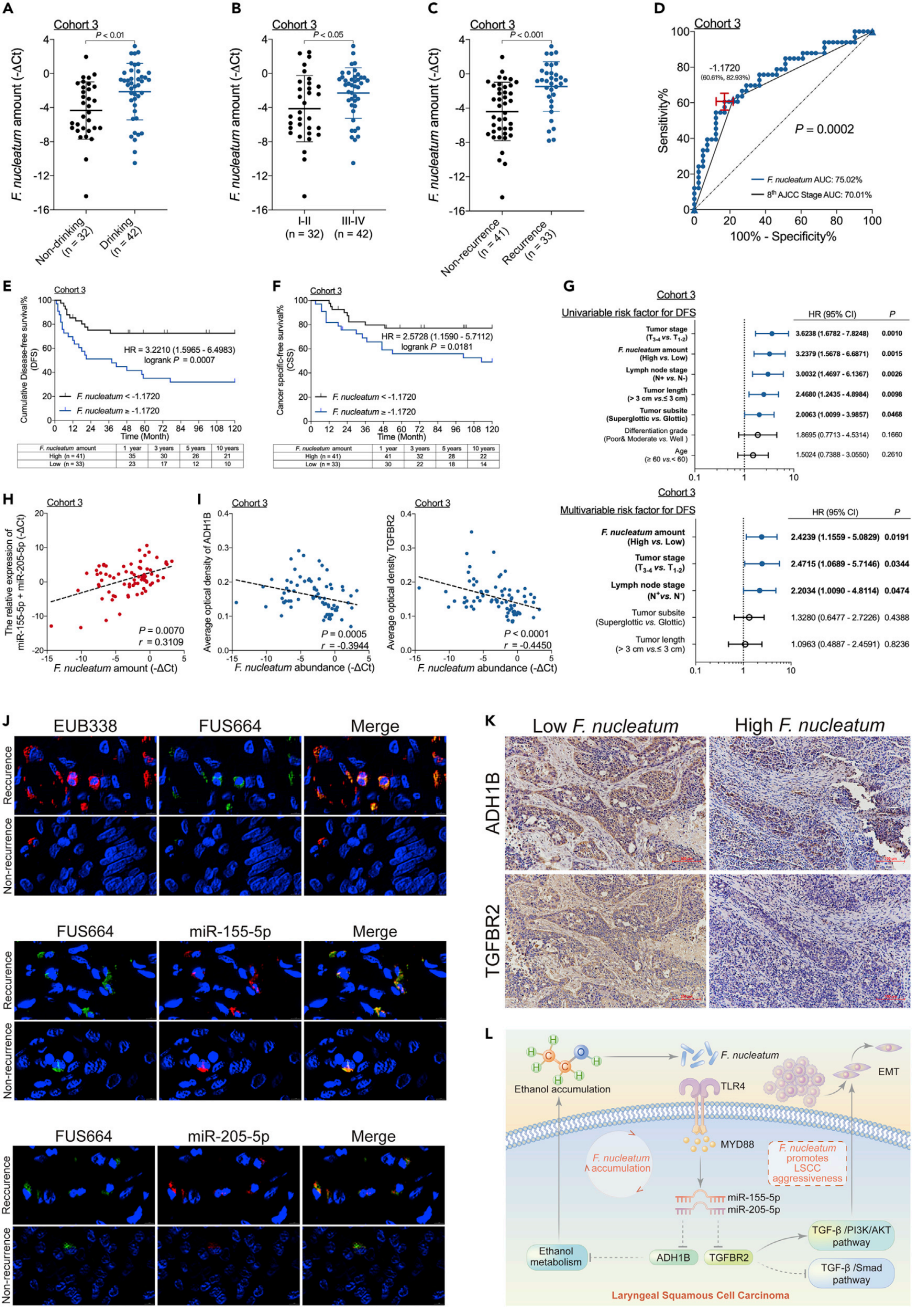
The *F. nucleatum* content correlates with the miR-155-5p, miR-205-5p, ADH1B, and TGFBR2 levels in LSCC and indicates poor oncologic outcome (A–C) Statistical analysis of the *F. nucleatum* amount in samples from patients in Cohort 3 as calculated by the Mann-Whitney test. (D) Receiver operating characteristic (ROC) analysis was conducted based on the amount of *F. nucleatum* and the eighth AJCC criteria for LSCC in samples from patients in Cohort 3. (E) DFS was determined in patients with LSCC from Cohort 3, who were stratified by high or low *F. nucleatum* content based on the cutoff value as assessed by the log rank test. (F) CSS was determined in patients with LSCC from Cohort 3, who were stratified by high or low *F. nucleatum* content based on the cutoff value as assessed by the log rank test. (G) Univariate analysis (top) and multivariate analysis (down) for DFS were performed on patients from Cohort 3. The bars correspond to 95% confidence intervals. (H) The correlation between *F. nucleatum* content and the combined expression level of miR-155-5p and miR-205-5p as assessed by the two-tailed nonparametric Spearman correlation coefficient. (I) The correlation between *F. nucleatum* content and ADH1B (left) and TGFFBR2 (right) expression in patients from Cohort 3 as assessed by the two-tailed nonparametric Spearman correlation coefficient. (J) Representative FISH assays (400 × magnification) to identify common bacteria-specific 16S rRNA sequences (EUB338, Cy3-labeled), *F. nucleatum* (FUS664, FAM-labeled), miR-155-5p (Cy3-labeled), and miR-205-5p (Cy3-labeled) in recurrent and nonrecurrent LSCC tissues. Scale bar, 2 μm. (K) Representative immunohistochemistry (10 × magnification) of ADH1B and TGFBR2 expression in the high and low *F. nucleatum* groups. Scale bar, 100 μm. (L) Schematic diagram of the relationship among *F. nucleatum*, ethanol metabolism reprogramming, and EMT in LSCC. Data are represented as mean ± SD.

To confirm and validate the clinical significance of *F. nucleatum*, we studied formalin-fixed, paraffin-embedded (FFPE) tissues from patients with LSCC in a third cohort (Cohort 3). The amount of *F. nucleatum* in LSCC was higher in patients with a history of alcohol consumption than in those without a history of alcohol consumption (Figure 6A). Moreover, the amount of *F. nucleatum* was higher in patients with advanced-stage LSCC than in those with early-stage LSCC (Figure 6B).

We next explored the relationship between the amount of *F. nucleatum* and LSCC recurrence in patients from Cohort 3. The amount of *F. nucleatum* in LSCC was higher in patients with recurrence than in patients without recurrence (Figure 6C and Table S4), and the area under the curve was higher for the *F. nucleatum*-based model than for the AJCC stage-based model (Figure 6D). The Youden index was used to determine the optimal cutoff value, and a -ΔCt value of 1.1720 was selected based on the amount of *F. nucleatum* that provided the best balance between the sensitivity and the specificity for predicting LSCC recurrence. Consistent with this result, a high level of *F. nucleatum* content was strongly associated with shorter DFS and CSS in patients with LSCC, in contrast with the survival rates in patients with a low level of *F. nucleatum* content (Figures 6E and 6F). In addition, *F. nucleatum* content was an independent predictor for DFS (Figure 6G) with a significant hazard ratio. Thus, the amount of *F. nucleatum* strongly affects LSCC progression and patient outcomes.

To investigate the clinical significance of *F. nucleatum* content and the expression levels of miRNAs (miR-155-5p and miR-205-5p), ADH1B, and TGFBR2, we analyzed FFPE LSCC tissues from patients in Cohort 3. The amount of *F. nucleatum* in LSCC tissues was positively correlated with the expression levels of miR-155-5p and miR-205-5p (Figure 6H) and negatively correlated with those of ADH1B and TGFBR2 (Figure 6I). Consistent with this result, FISH showed that *F. nucleatum* promoted miR-155-5p and miR-205-5p expression in patients with LSCC with recurrence (Figure 6J). Moreover, IHC staining showed that increased *F. nucleatum* content decreased ADH1B and TGFBR2 expression (Figure 6K). Considering these findings collectively, it is reasonable to speculate that *F. nucleatum* promotes LSCC aggressiveness via the TLR4/MYD88 pathway, leading to the suppression of ADH1B and TGFBR2 expression via selective gain of miR-155-5p and miR-205-5p and consequently resulting in ethanol metabolism reprogramming and EMT in LSCC (Figure 6L).

## Discussion

The study aimed to assess the importance of *F. nucleatum* in LSCC progression and prognosis, and elevated *F. nucleatum* content was found to lead to treatment failure and poor prognosis in patients with LSCC. *F. nucleatum*, a gram-negative anaerobic bacterium, is a common component of the oral microbiota and has long been considered to cause periodontitis (Han and Wang, 2013). Recent studies have indicated that a high amount of *F. nucleatum* is closely associated with poor prognosis in patients with alimentary cancers (Serna et al., 2020; Yamamura et al., 2019; Yang et al., 2017; Yu et al., 2017). Our data revealed that the amount of *F. nucleatum* is an independent risk factor for LSCC recurrence and should be considered a significant indicator of poor prognosis. Although the amount of *F. nucleatum* was higher in LSCC tissues than in non-LSCC tissues, the extent to which *F. nucleatum* acts as a carcinogenic microorganism in LSCC remains unclear. Thus, elucidating the function and mechanism of *F. nucleatum* is essential for optimizing the current therapeutic strategies to improve prognosis by preventing LSCC progression.

Alcohol consumption is a common addictive behavior and an important prognostic factor in LSCC (Giraldi et al., 2017). When matching for treatment and primary site, patients with LSCC with a history of heavy alcohol consumption have a significantly worse prognosis than do patients with no history of alcohol consumption (Sawabe et al., 2017). Moreover, alcohol abstinence was found to be associated with a yearly reduction of 2% in the risk of laryngeal cancer, and the time of abstinence until the risk was equal to that of individuals with no history of alcohol consumption was 36 years (Ahmad Kiadaliri et al., 2013). Consistent with the present results, previous studies have demonstrated that the amount of *F. nucleatum* is significantly higher in individuals who engage in heavy alcohol consumption (Kim et al., 2020; Lages et al., 2015).

Host cell-dependent aerobic growth of *F. nucleatum* was observed in 12 colon cancer cell lines (Okita et al., 2020); therefore, it can be assumed that *F. nucleatum* and ethanol metabolism are closely related. Previous studies of ethanol-metabolizing capacity have typically focused on genetic polymorphisms in enzymes related to ethanol metabolism in HNSCC (Chang et al., 2012; Koyanagi et al., 2020). ADH1B belongs to the ADH family and has been identified as a tumor suppressor enzyme in HNSCC (Chang et al., 2012). Our results revealed that *F. nucleatum* inhibits ethanol metabolism by downregulating ADH1B expression, which ultimately leads to ethanol accumulation. Moreover, local accumulation of ethanol promotes *F. nucleatum* proliferation. Thus, the interaction between *F. nucleatum* and ethanol metabolism reprogramming forms a feed-forward loop, which may account for the increased *F. nucleatum* content in LSCC.

In the present study, *F. nucleatum* content was higher in patients with advanced-stage LSCC than in those with early-stage LSCC. Moreover, *F. nucleatum* promoted the migration, invasion, and lung metastasis of LSCC cells. One unexpected finding was the extent to which *F. nucleatum* promoted neck and thigh metastasis in the experimental lung metastasis model. These results are consistent with the findings of other studies showing that *F. nucleatum* promotes tumor progression in alimentary and respiratory cancers (Harrandah et al., 2020; Yu et al., 2020). In epithelium-derived solid malignancies, EMT is a key step in LSCC invasion and metastasis and is a leading cause of poor prognosis (Wang et al., 2020). *F. nucleatum* promotes EMT by regulating the expression of E-cadherin, Vimentin, N-cadherin, and Snail1 in noncancerous human immortalized oral epithelial cells and oral squamous cell cancer (Zhang et al., 2020) and by regulating the expression of Snail, E-cadherin, and fibronectin in colorectal cancer (Yu et al., 2020). TGF-β plays a dual role in cancer progression (Ahn et al., 2018). In the early stage, the TGF-β signaling pathway induces cell-cycle arrest and apoptosis to prevent cancer progression. However, in advanced-stage LSCC, the TGF-β signaling pathway acts as a key promoter of cancer progression and metastasis without genetic inactivation of the TGF-β receptor (Gu and Feng, 2018). TGFBR2 is an important component of the TGF-β signaling pathway, and loss or reduced expression of TGFBR2 may abrogate TGF-β signaling to promote tumor progression (Lu et al., 2006; Nerlich et al., 2003). Indeed, our results revealed that *F. nucleatum* promotes EMT in LSCC by suppressing TGFBR2 expression, which is in agreement with the findings of other studies showing that inhibiting TGFBR2 expression results in activation of the PI3K/AKT pathway (Lyu et al., 2014).

*F. nucleatum* lipopolysaccharide leads to strong induction of the TLR4/MYD88 innate immune signaling pathway to regulate miRNA expression (Liu et al., 2007), and the expression of specific miRNAs can promote tumor proliferation and the development of chemoresistance through the TLR4/MYD88 pathway (Yang et al., 2017; Yu et al., 2017). Our previous study demonstrated that miR-155-5p and miR-205-5p expression was higher in LSCC tissues than in non-LSCC tissues; these miRNAs may play important roles in LSCC initiation and progression (Cao et al., 2013). Moreover, the expression of miR-155-5p and miR-205-5p was reported to be closely related to EMT in LSCC (Cui et al., 2019; Zhao et al., 2017). Our bioinformatic and functional studies elucidated that miR-155-5p and miR-205-5p target ADH1B and TGFBR2, respectively. These results are consistent with those of recent studies showing that *F. nucleatum* increases the expression of miR-155-5p and miR-205-5p through the TLR4/MYD88 pathway, resulting in ethanol metabolism reprogramming via suppression of TGFBR2 expression and subsequent suppression of ADH1B expression and EMT in LSCC.

In conclusion, the current findings clearly indicate that *F. nucleatum* suppresses ADH1B expression, resulting in ethanol metabolism reprogramming. Accumulated ethanol promotes *F. nucleatum* proliferation, ultimately resulting in an inability to alleviate ethanol-induced metabolic stress in LSCC. Moreover, *F. nucleatum* promotes cell proliferation, migration, and invasion in LSCC, and the interaction between *F. nucleatum* and ethanol further enhances the aggressiveness of LSCC. *F. nucleatum* suppresses TGFBR2, resulting in EMT in LSCC by increasing N-cadherin, Vimentin, and ZEB1 expression while decreasing E-cadherin expression via attenuation of the Smad-dependent TGF-β signaling and activation of PI3K/AKT via the Smad-independent TGF-β signaling pathway*. F. nucleatum* increases the expression of miR-155-5p and miR-205-5p by activating MYD88-dependent TLR4 signaling in LSCC, resulting in ethanol metabolism reprogramming via ADH1B and EMT via TGFBR2. The clinical results presented here also highlight the identification of *F. nucleatum* as a risk factor for recurrence in patients with LSCC. Taken together, our data yield valuable insight into the positive feed-forward loop between *F. nucleatum* and ethanol metabolism reprogramming that promotes LSCC cell invasion and metastasis to affect the prognosis of patients with LSCC.

### Limitations of the study

In addition to confirming the importance of the mechanism linking *F. nucleatum* with LSCC, further studies should be conducted with a larger sample size and should follow long-term LSCC patients to fully validate our results. Moreover, further studies exploring the possibility of reducing the *F. nucleatum* content to prevent local relapse and metastasis of LSCC should be undertaken.

## STAR★Methods

### Key resources table

**Table:** 

REAGENTS or RESOURCES	SOURCE	IDENTIFIER
**Antibodies**		

Anti-ADH1B	Proteintech	Cat#66939-1-Ig; RRID: AB_2882263
Anti-TGFBR2	Abcam	Cat#ab186838; RRID: AB_2728775
Anti-E-cadherin	Cell Signaling Technology	Cat#3195; RRID: AB_2291471
Anti-N-cadherin	Cell Signaling Technology	Cat#13116; RRID: AB_2687616
Anti-Vimentin	Abcam	Cat# ab92547; RRID: AB_10562134
Anti-ZEB-1	Cell Signaling Technology	Cat#3396; RRID: AB_1904164
Anti-Smad2/3	Abclonal	Cat#A18674; RRID: AB_2862410
Anti-p-Smad2/3	Abclonal	Cat#AP0548; RRID: AB_2771541
Anti-PIK3CA	Abclonal	Cat#A0265; RRID: AB_2757078
Anti-p-Akt	Abclonal	Cat#AP0637; RRID: AB_2770898
Anti-TLR4	Abclonal	Cat#A5258; RRID: AB_2766084
Anti-MYD88	Proteintech	Cat#23230-1-AP; RRID: AB_2879236
Anti-GAPDH	Abcam	Cat#ab181602; RRID: AB_2630358

**Biological samples**		

Fresh LSCC tissues	Eye & ENT Hospital, Fudan University	N/A
Para-cancerous normal epithelial cell tissues	Eye & ENT Hospital, Fudan University	N/A
Formalin-fixed paraffin-embedded LSCC tissues	Eye & ENT Hospital, Fudan University	N/A

**Critical commercial assays**		

QIAamp BiOstic Bacteremia DNA Kit	Qiagen	Cat#12240–50
Miscript II RT kit	Qiagen	Cat#218161
QIAamp DNA FFPE Tissue Kit	Qiagen	Cat#56404
QuantiNova SYBR Green RT-PCR Kit	Qiagen	Cat#208154
miScript SYBR Green PCR Kit	Qiagen	Cat#218075
Ethanol Assay Kit	Abcam	Cat#ab65343
Aldehyde Assay Kit	Abcam	Cat#ab112113
Alcohol Dehydrogenase Assay Kit	Abcam	Cat#ab102533
Dual Luciferase Reporter Gene Assay Kit	Yeasen	Cat#11402ES60
RiboTM Fluorescent *In Situ* Hybridization Kit	Ribobio	Cat#C10910

**Experimental models: Organisms/strains**		

*Fusobacterium nucleatum* strain 25586	ATCC	Cat#59899827
*Escherichia coli* strain DH5a	TIANGEN	Cat#CB101
Sequences of miRNA mimics, miRNA inhibitors, and mRNA siRNAs, See Table S5	Sangon Biotech	N/A
DNA primer sequences, See Table S5	Sangon Biotech	N/A
miRNA and U6 primer sequences, See Table S5	Sangon Biotech	N/A

**Deposited data**		

Raw images for all western blots	Mendeley Data	https://data.mendeley.com/datasets/c7wztxntdx/1

**Experimental models: Cell lines**		

AMC-HN-8	Eye & ENT Hospital, Fudan University	CVCL_5966
FD-LSC-1	Eye & ENT Hospital, Fudan University	CVCL_S891
LSCC-31-C3	Eye & ENT Hospital, Fudan University	N/A

**Software and algorithms**		

SPSS	IBM	https://www.ibm.com/analytics/spss-statistics-software
R	R Development Core Team	https://www.r-project.org/
Prism	GraphPad	https://www.graphpad.com/scientific-software/prism/
CaseViewer	3DHISTECH	https://www.3dhistech.com/solutions/caseviewer/
Adobe Illustrator	Adobe	https://www.adobe.com/products/illustrator.html

### Resource availability

#### Lead contact

Further information and requests for resources and reagents should be directed to the lead contact Liang Zhou (zhoulent@126.com).

#### Materials availability

This study did not generate new unique reagents.

### Experimental model and subject details

#### Mice

For the xenograft experiments, six-week-old male BALB/c nude mice (Shanghai Laboratory Animal Company, Shanghai, China) were housed in laminar flow cabinets under specific pathogen-free conditions with autoclaved food and water provided *ad libitum*.

To establish the xenograft model, 8 × 10^6^ AMC-HN-8 cells with or without stable transduction of the lentiviral overexpression vector containing TGFBR2 overexpression vector were subsequently cocultured with *F. nucleatum*. These treated cells were then suspended in 100 μL of PBS (PBS) and subcutaneously injected into the right flank of the nude mice (5 mice per group). Tumors were monitored at set times, and tumor volumes were calculated as follows: Volume = (length × width^2^)/2. The mice were euthanized on day 33, and the tumors were weighed.

In the metastasis experiment, 1 × 10^6^ AMC-HN-8 cells were suspended in 100 μL of PBS and subcutaneously injected into six-week-old nude mice via tail vein. After 8 weeks, the mice were euthanized, and suspected tumor nodules in the lung and other areas of the body were fixed with 4% (w/v) paraformaldehyde and embedded in paraffin. Paraffin sections (5 μm thick) were stained with hematoxylin-eosin (HE) to calculate the number of microscopic metastatic nodules. All animal experiments were approved by the Animal Center at the Eye & ENT Hospital, Fudan University.

#### Bacterial culture

The *F. nucleatum* strain added to the cell cultures at a human cell:bacterial cell ratio of 1:100. *F. nucleatum* were grown in Columbia blood agar supplemented with 5 mg/mL hemin, 5% defibrinated sheep blood, and 1 mg/mL vitamin K1 and cultured overnight at 37°C under anaerobic conditions. The commensal *E. coli strain DH5a* propagated in Difco Luria-Bertani medium (BD Biosciences, California, USA) and cultured overnight at 37°C under anaerobic conditions.

#### Cell culture

Three human LSCC cell lines (AMC-HN-8, FD-LSC-1, and LSCC-31-C3) were used in this study. AMC-HN-8 cells were cultured in RPMI-1640 medium (HyClone, Utah, USA), whereas FD-LSC-1 and LSCC-31-C3 cells were cultured in BEGM™ Bronchial Epithelial Cell Growth Medium (Lonza, Maryland, USA) supplemented with 1% (v/v) penicillin-streptomycin and 10% fetal bovine serum (FBS). All cells were maintained at 37°C in a humidified atmosphere containing 5% CO_2_.

#### Patients and specimen collection

This study included three cohorts comprising a total of 245 LSCC patients and 65 vocal cord polyp patients at the Eye & ENT Hospital, Fudan University between 2009 and 2020. Tissues from patients in Cohort one comprised 131 pairs of CLC and PLC as well as 35 Controls and were obtained between 2017 and 2020 (Table S1); tissues from patients in Cohort two comprised 40 CLC, 40 PLC, and 30 control tissues obtained between 2009 and 2010 (Table S2); and tissues from patients in Cohort three comprised 74 FFPE sections of LSCC tissues obtained between 2009 and 2010 (Table S4).

The inclusion criteria were as follows: (i) presence of a signature from an LSCC patient on an informed consent form obtained before surgery and (ii) confirmation of LSCC and classification of tumor stage according to the eighth edition of the AJCC cancer staging manual by experienced pathologists and complete clinical, imaging, laboratory, and pathological data. The exclusion criteria were as follows: (i) histopathological confirmation of multiple types of primary LSCC, (ii) active bacterial or viral infection, (iii) preoperative treatment with regimens such as radiotherapy or chemotherapy, (iv) use of antibiotics during the previous 3 months, or (v) history of autoimmune disease or treatment with steroids.

The patient demographic data collected included sex, age, hypertension status, diabetes status, smoking index (Sulsky et al., 2014), and drinking index (Hsueh et al., 2020). The clinical characteristics of LSCC patients included tumor subsite, tumor length, local and regional extension category of the primary tumor, TNM stage, and pathological differentiation status.

### Method details

#### Bacterial colony formation assays

To assess the colony formation capacity of *F. nucleatum*, colonies were cultured in Columbia blood agar in the presence of ethanol at 37°C under anaerobic conditions for 72 h. The number of *F. nucleatum* colonies was counted in triplicate for each group.

#### Detection of *F. nucleatum*

The cycle threshold (Ct) value for *F. nucleatum* DNA normalized to the amount of genomic DNA (gDNA) in human biopsy tissue was determined by using the primer set for the reference gene prostaglandin transporter (PGT) as previously described (Castellarin et al., 2012). gDNA was extracted from fresh tissues and from FFPE tissues. Amplification and detection of *F. nucleatum* DNA were conducted in a 96-well optical PCR plate with an ABI 7500 Real-Time PCR System (Thermo Fisher, Massachusetts, USA) as described previously (Hsueh et al., 2020). The Ct values obtained from the samples were compared using the-ΔCt method.

#### Proliferation, migration, and invasion assays

Proliferation, migration, and invasion assays are commonly used to evaluate the aggressiveness of cancers. Cell proliferation was assayed using a Cell Counting Kit-8 (CCK8, Dojindo, Kumamoto, Japan). For migration assays, 1 × 10^6^ LSCC cells cultured in the presence or absence of *F. nucleatum* and 0.1% (v/v) ethanol for 72 h were resuspended in serum-free medium and seeded into the upper chamber of Transwell inserts (Corning, New York, USA); the membrane pore size was 8 μm. RPMI 1640 or BEGM supplemented with 10% FBS was added to the bottom chamber. After 24 h of incubation, the inserts were removed, washed with PBS, fixed with 4% methanol, and then stained with crystal violet. The numbers of stained cells were counted in three fields/insert under a light microscope. For invasion assays, the inserts of the chambers in which the cells were seeded were coated with Matrigel (BD Biosciences).

#### Determination of ethanol and acetaldehyde concentrations

The ethanol and acetaldehyde concentrations in the LSCC cell lines cultured in the presence or absence of *F. nucleatum* and 0.1% (v/v) ethanol for 72 h were measured and quantified according to the manufacturer’s instructions.

#### RNA extraction and measurement

Total RNA was extracted from tissue samples and LSCC cells using TRIzol reagent (Thermo Fisher). Total cDNA was reverse transcribed from 1 μg of total RNA. Relative miRNA and mRNA levels were determined according to the manufacturer’s instructions.

The quantitative real-time PCR assay was conducted in triplicate on an ABI 7500 Real-Time PCR system (Thermo Fisher). The Ct values obtained from the different samples were compared using the-ΔCt method. The housekeeping genes GAPDH and U6 were used as internal references to normalize gene expression for mRNA and miRNA, respectively.

#### Western blot analysis, immunohistochemistry, immunofluorescence and enzyme linked immunosorbent assay

For western blot analysis, LSCC tissues or cells were lysed in RIPA lysis buffer (Beyotime, Shanghai, China) supplemented with a protease inhibitor and 1 mM phenylmethanesulfonylfluoride (PMSF, Beyotime) for protein extraction. Thirty micrograms of protein were electrophoresed via SDS–PAGE through 4–20% gels and transferred onto nitrocellulose membranes. The membranes were blocked with 5% BSA (BSA, Yeasen, Shanghai, China) and incubated with the primary and secondary antibodies according to the manufacturer’s instructions. Immunoreactions were detected using enhanced chemiluminescence (ECL). An anti-GAPDH antibody was used as the internal control to normalize the data.

For IHC, FFPE LSCC tissue samples were sliced into 5-μm-thick sections and incubated with specific primary antibodies overnight at 4°C. The following day, the sections were washed twice and incubated with a horseradish peroxidase (HRP)-conjugated secondary antibody at room temperature, after which they were stained with 3,3′-diaminobenzidine (DAB) solution and hematoxylin and evaluated under a light microscope.

For IF assays, LSCC cells were seeded on slides at the appropriate cell density, maintained at 37°C overnight and then incubated with a fluorescent secondary antibody (Yeasen, Shanghai, China) prior to the addition of 4′,6-diamidino-2-phenylindole (DAPI, Thermo Fisher). Cells were fixed with 4% (w/v) paraformaldehyde for 15 min and permeabilized with 0.5% (v/v) Triton X-100 (Thermo Fisher) for 5 min prior to blocking with 5% (w/v) BSA for 1 h. After the final treatment, labeled cells were examined using an ECLIPSE Ts2R fluorescence imaging microscope (Nikon, Tokyo, Japan).

For the ELISA, ADH activity in LSCC cells was measured and quantified using an Alcohol Dehydrogenase Assay Kit (Abcam).

#### Fluorescence in situ hybridization

Localization of *F. nucleatum*, miR-155-5p, and miR-205-5p in LSCC tissues was evaluated by FISH on 5-μm-thick FFPE sections using a Fluorescence *In Situ* Hybridization Kit (RiboBio, Guangzhou, China). For the detection of 16S rRNA-targeted probes, the sequence was obtained from probeBase (http://www.microbial-ecology.net/probebase/) and synthesized (Generay, Shanghai, China). The sequence of “the universal bacterial” probe was EUB338 (Cy3-labeled) (Tokar et al., 2018), and the sequence of the *F. nucleatum*-targeted probe was FUS664 (FAM-labeled) (Yu et al., 2016).

#### Dual-luciferase assay

HEK293T and LSCC cells were transfected with synthetic miR-155-5p and miR-205-5p mimics and cotransfected with wild-type or mutant pGL3 plasmid and the firefly luciferase reporter plasmid using Lipofectamine 2000 reagent (Thermo Fisher). Twenty-four hours after transfection, the firefly and Renilla luciferase activities were quantified using a Dual Luciferase Reporter Assay Kit (Yeasen). The transfection efficiency data were normalized by dividing the firefly luciferase activity and the Renilla luciferase activity. Each transfection was performed independently in triplicate.

### Quantification and statistical analyses

#### Statistical analysis

Pearson’s chi-squared test or Fisher’s exact test was used to analyze associations with clinicopathological parameters as appropriate. Differences in quantitative data between two groups were evaluated using unpaired or paired Student’s t test, the Wilcoxon matched pairs signed-rank test, or the Mann-Whitney U test as appropriate. Comparisons of means among multiple groups were performed with ordinary one-way ANOVA or ordinary two-way ANOVA. The relationships between the *F. nucleatum* content and the expression levels of miRNAs and mRNAs were analyzed via the two-tailed nonparametric Spearman correlation coefficient. The impacts of clinical parameters were estimated by using univariate or multivariate CPH regression analysis. Receiver operating characteristic (ROC) analysis was performed to identify the sensitivity and specificity of *F. nucleatum* content and the optimal cutoff value for predicting LSCC. The patient clinical endpoints were calculated using the Kaplan-Meier method and compared with the log rank test. All p values were two-tailed, and p values <0.05 were considered to indicate significant differences. All statistical analyses were performed using GraphPad Prism nine software (California, USA) and IBM SPSS Statistics software (Illinois, USA).

## Data Availability

• Original Western blot images have been deposited at Mendeley and are publicly available as of the date of publication at Mendeley data: https://data.mendeley.com/datasets/c7wztxntdx/1. • This paper does not report original code. • Any additional information required to reanalyze the data reported in this paper is available from the lead contact upon request.
