# Supplementary material for: A positive feed-forward loop between Fusobacteriumnucleatum and ethanol metabolism reprogramming drives laryngeal cancer progression and metastasis
Source: iScience. 2022 Jan 30;25(2):103829. doi: 10.1016/j.isci.2022.103829 (PMC8851092; doi:10.1016/j.isci.2022.103829)
Supplement: Document S1. Figures S1–S8 and Tables S1–S5 [file mmc1.pdf]

iScience, Volume *25*

Supplemental information

A positive feed-forward loop between *Fusobacterium nucleatum* and ethanol metabolism reprogramming drives laryngeal cancer progression and metastasis

Chi-Yao Hsueh, Qiang Huang, Hongli Gong, Yujie Shen, Ji Sun, Hui-Ching Lau, Duo Zhang, Di Tang, Chunping Wu, Yang Guo, Huiying Huang, Pengyu Cao, Lei Tao, Ming Zhang, and Liang Zhou

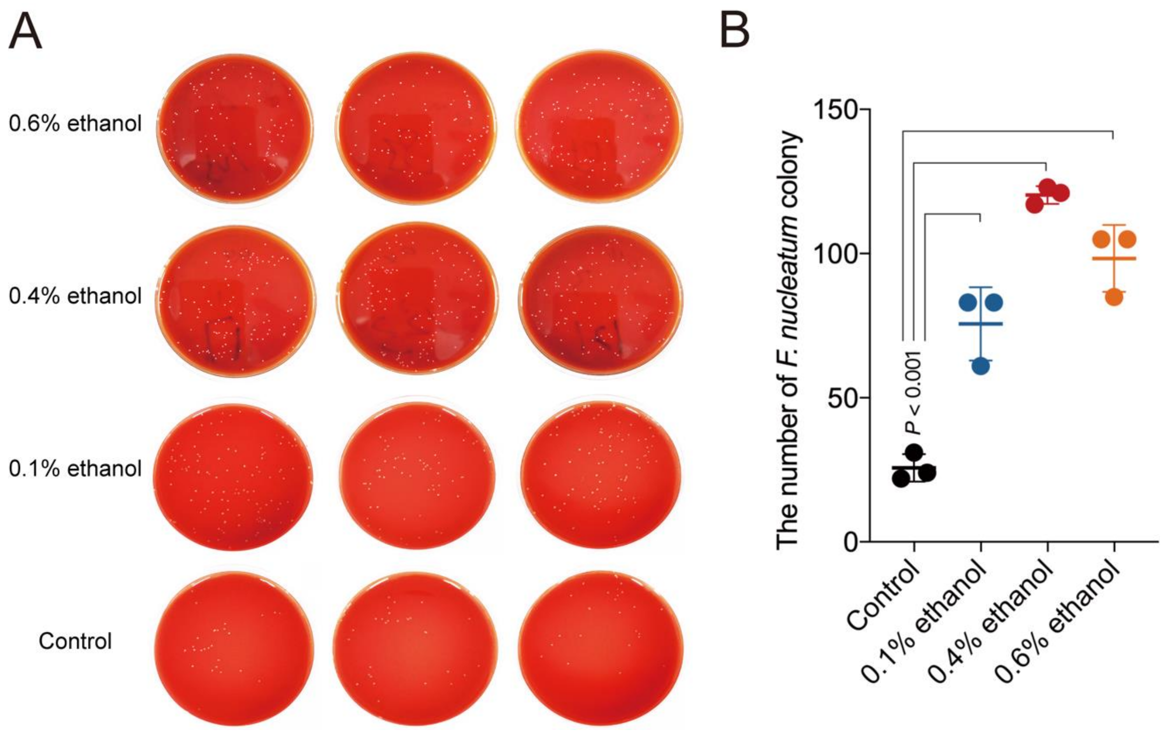

Figure S1. Ethanol promotes *F. nucleatum* proliferation, related to Figure 2.

(A) Representative data of *F. nucleatum* colony in 0.1%, 0.4% and 0.6% (v/v) ethanol by colony formation under different experimental conditions (n = 3 for each group).

(B) Statistical analysis of the number of *F. nucleatum* colony to different experimental conditions (n = 3 for each group) as assessed by the ordinary two-way ANOVA. Data are represented as mean ± SD.

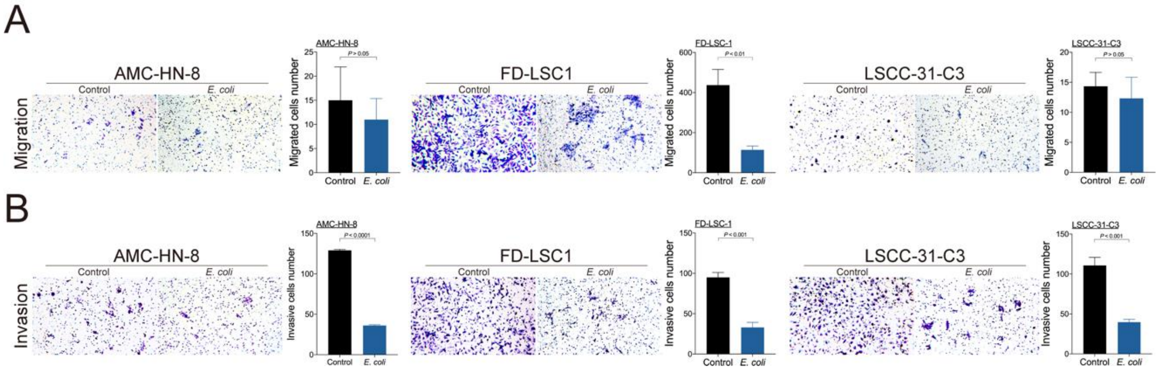

Figure S2. ***E. coli*** **does not promote cell migration and invasion in LSCC, related to Figure 2.**

(A) Transwell migration assays were performed in 3 LSCC cell lines cultured in the presence or absence of *E. coli strain DH5a* as calculated with the Student's unpaired t test.

(B) Transwell invasion assays were performed in 3 LSCC cell lines cultured in the presence or absence of *E. coli strain DH5a* as calculated with the Student's unpaired t test. Data are represented as mean ± SD.

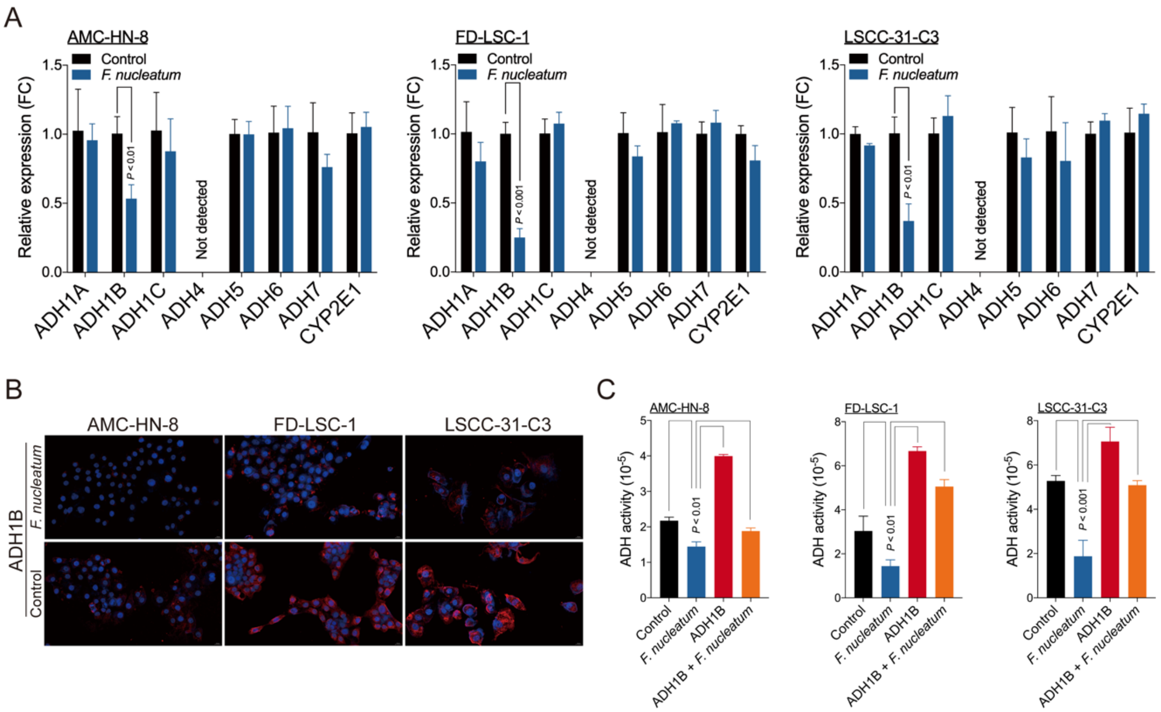

Figure S3. *F. nucleatum* inhibits ADH1B expression, related to Figure 2.

(A) Statistical analysis of the relative expression of 7 members of ADH family and CYP2E1 in 3 LSCC cell lines cultured in the presence or absence of *F. nucleatum* as assessed by Students unpaired t-test.

(B) Representative immunofluorescence was showed (40 × magnification) on ADH1B expression in 3 LSCC cell lines in the presence or absence of *F. nucleatum*. Bar scale, 20 μm.

(C) The ADH enzymatic activity was measured by ELISA assay in 3 LSCC cell lines with or without ADH1B-overexpressing lentiviral vector transduction and subsequently cultured with *F. nucleatum* as assessed by the ordinary one-way ANOVA. Data are represented as mean ± SD.

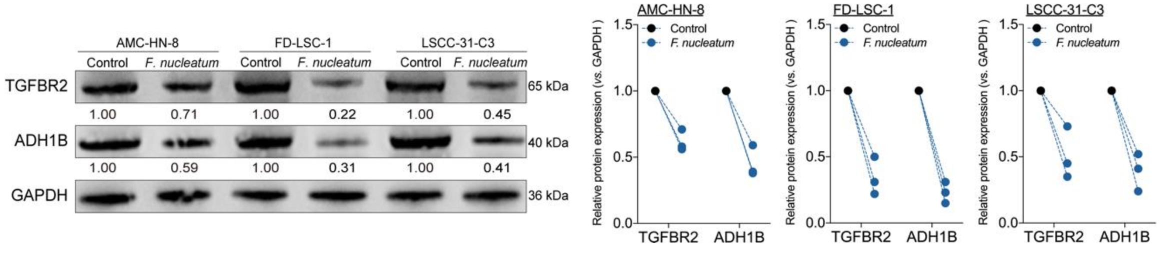

Figure S4. *F. nucleatum* inhibits ADH1B and TGFBR2 expression, related to Figure 2, 3.

Western blotting was performed to assess ADH1B and TGFBR2 expression in 3 LSCC cell lines cultured with *F. nucleatum*.

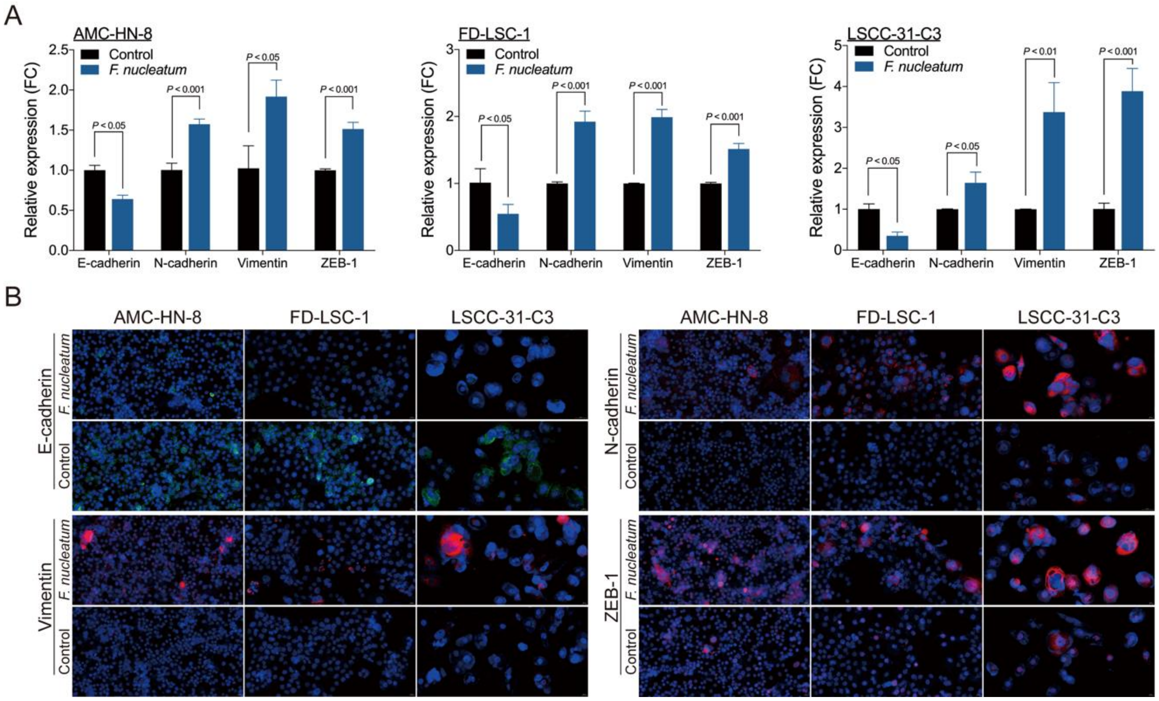

Figure S5. *F. nucleatum* promotes EMT in LSCC, related to Figure 3.

(A) Statistical analysis of the relative expression of EMT-related genes (E-cadherin, N-cadherin, Vimentin, and ZEB-1) in 3 LSCC cell lines cultured in the presence or absence of *F. nucleatum* as assessed by Students unpaired t-test.

(B) Representative immunofluorescence was showed (40 × magnification) on E-cadherin, N-cadherin, Vimentin, and ZEB-1 expression in 3 LSCC cell lines in the presence or absence of *F. nucleatum*. Bar scale, 20 μm. Data are represented as mean ± SD.

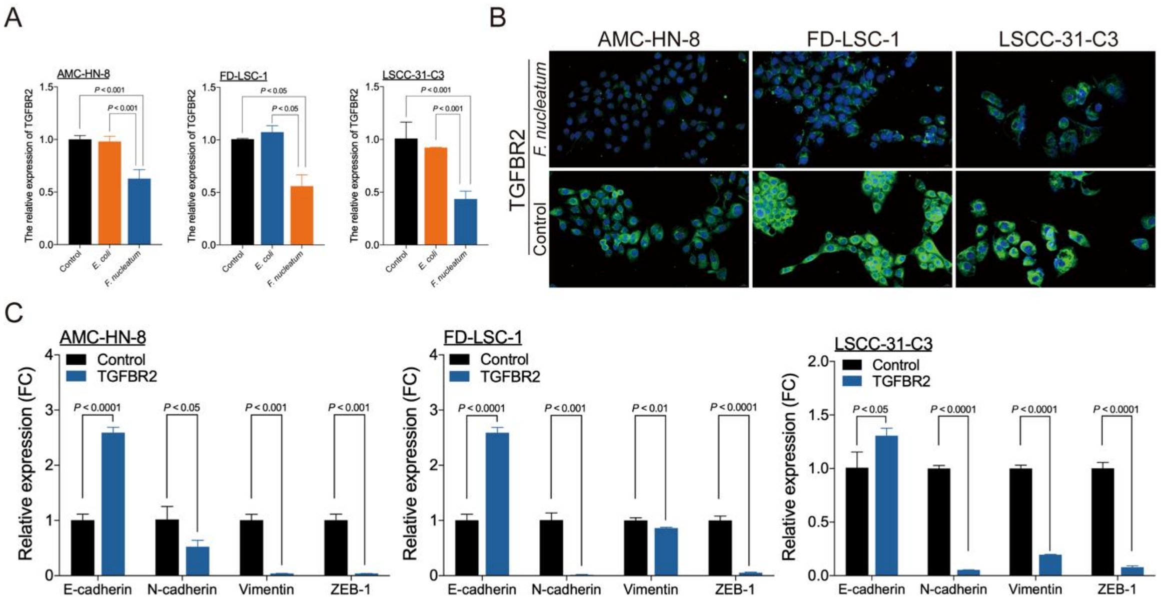

Figure S6. *F. nucleatum*-induced EMT in LSCC by suppressing TGFBR2 expression, related to Figure 3.

(A) Statistical analysis of the relative expression of TGFBR2 in 3 LSCC cell lines cultured with *F. nucleatum* or *E. coli* as calculated with Students unpaired t-test.

(B) Representative immunofluorescence was showed (40 × magnification) on TGFBR2 expression in 3 LSCC cell lines in the presence or absence of *F. nucleatum*. Bar scale, 20 μm.

(C) Statistical analysis of the relative expression of EMT-related genes (E-cadherin, N-cadherin, Vimentin, and ZEB-1) in 3 LSCC cell lines with or without TGFBR2 overexpressed lentiviral vector transduction as calculated with Students unpaired t-test. Data are represented as mean ± SD.

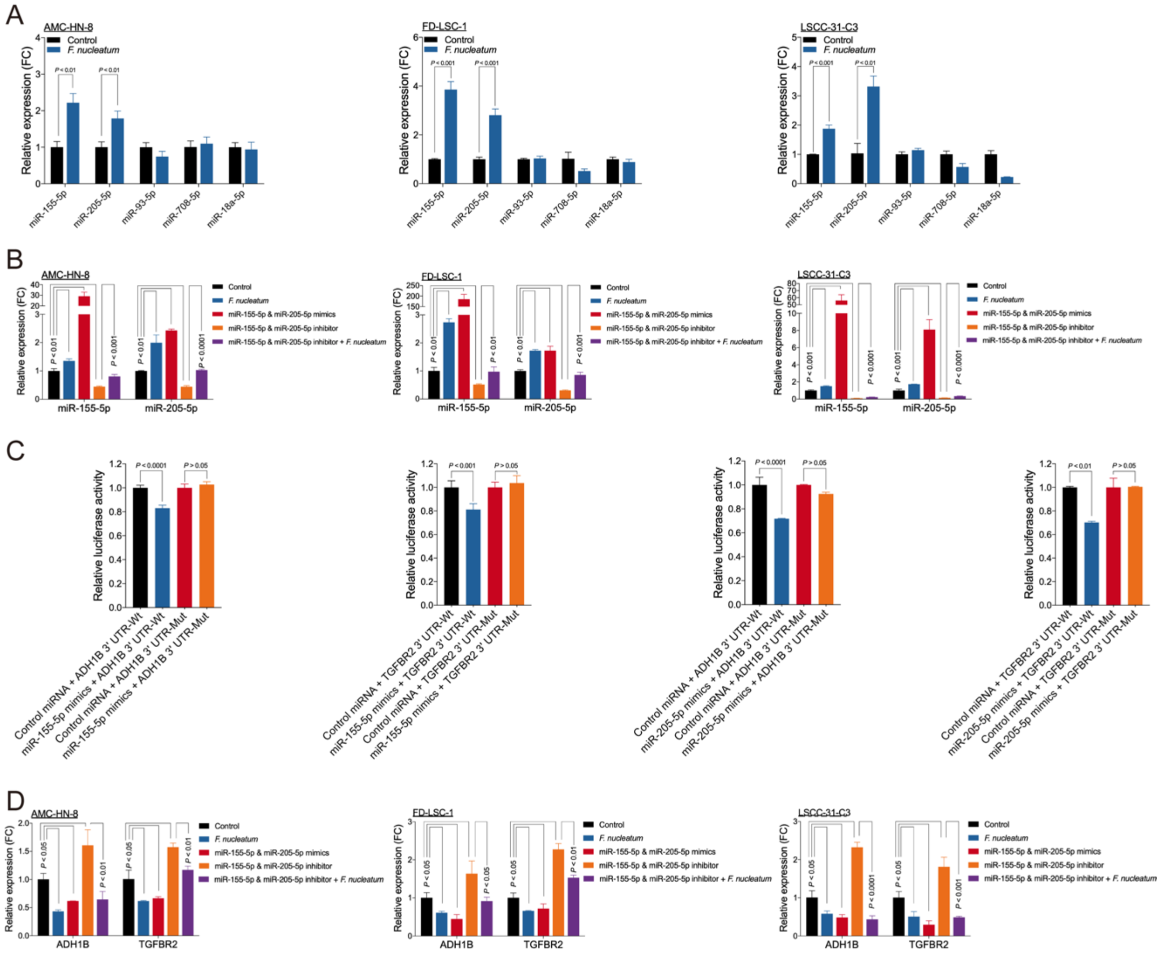

Figure S7. *F. nucleatum* inhibits ADH1B and TGFBR2 expression by increasing miR-155-5p and miR-205-5p, related to Figure 4.

(A) Statistical analysis of the relative expression of LSCC-related miRNAs in 3 LSCC cell lines transfected with miR-155-5p and miR-205-5p mimics or inhibitor and cultured in the presence or absence of *F. nucleatum* as calculated with Students unpaired t-test.

(B) Statistical analysis of the relative expression of miR-155-5p and miR-205-5p in 3 LSCC cell lines transfected with miR-155-5p and miR-205-5p mimics or inhibitor and cultured in the presence or absence of *F. nucleatum* as calculated with Students unpaired t-test.

(C) Dual-luciferase activity was measured in HEK293T cell line cultured treated with miR-155-5p & miR-205-5p mimics or control miRNA. Dual-luciferase reporters expressing wild-type (Wt) or mutant (Mut) 3'UTRs from human ADH1B and TGFBR2 mRNA were used.

The dual-luciferase activity was normalized based on the control miRNA transfection as assessed by the ordinary one-way ANOVA.

(D) Statistical analysis of the relative expression of ADH1B and TGFBR2 in 3 LSCC cell lines transfected with miR-155-5p and miR-205-5p mimics or inhibitor and cultured in the presence or absence of *F. nucleatum* as calculated with Students unpaired t-test. Data are represented as mean ± SD.

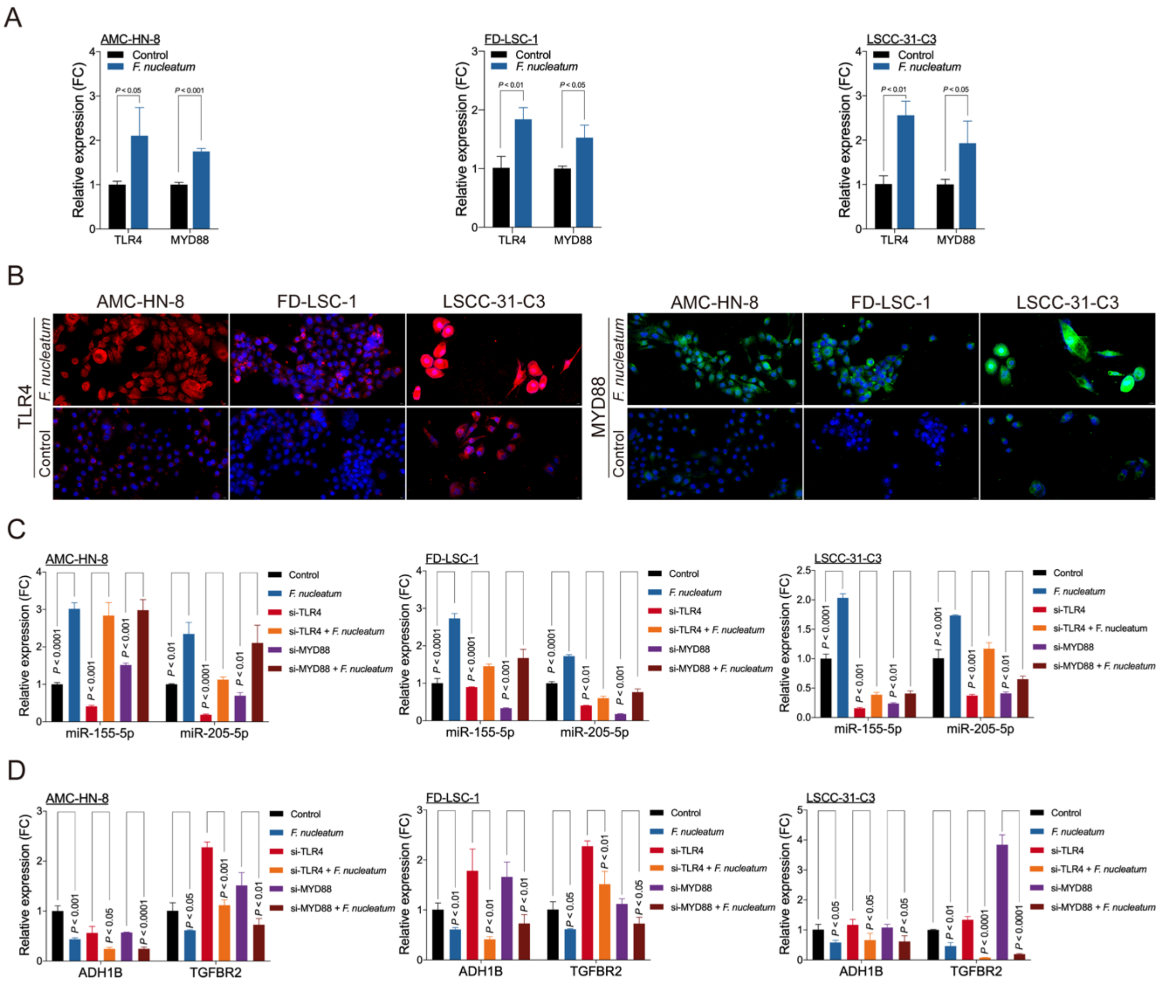

Figure S8. TLR4 and MYD88 are involved in *F. nucleatum*-mediated ADH1B and TGFBR2 expression, related to Figure 4.

(A) Statistical analysis of the relative expression of TLR4 and MYD88 in 3 LSCC cell lines cultured in the presence or absence of *F. nucleatum* as assessed by Students unpaired t-test.

(B) Representative immunofluorescence was showed (40 × magnification) on TLR4 and MYD88 expression in 3 LSCC cell lines in the presence or absence of *F. nucleatum*. Bar scale, 20 μm.

(C) Statistical analysis of the relative expression of miR-155-5p and miR-205-5p in 3 LSCC cell lines transfected with TLR4 and MYD88 siRNAs and cultured in the presence or absence of *F. nucleatum* as calculated with Students unpaired t-test.

(D) Statistical analysis of the relative expression of ADH1B and TGFBR2 in 3 LSCC cell lines transfected with TLR4 and MYD88 siRNAs and cultured in the presence or absence of *F. nucleatum* as calculated with Students unpaired t-test. Data are represented as mean ± SD.

Table S1. The Correlation of *F. nucleatum* content (-ΔCt) and clinicopathologic factors in Cohort 1, related to Figure 1.

Characteristics	Total, n	*F. nucleatum* amount (-ΔCt) (mean ± SD)	*P*
Age, years			0.1430
≥ 60	95	-7.7938 ± 4.8223	
< 60	36	-9.0972 ± 4.3039	
Sex			0.8450
Male	128	-8.1428 ± 4.7559	
Female	3	-8.5411 ± 1.6662	
Smoking history			0.4429
Yes	120	-8.2583 ± 4.6641	
No	11	-6.9917 ± 5.2320	
**Drinking history**			**0.0421**
**Yes**	**79**	**-7.3842 ± 5.1911**	
**No**	**52**	**-9.3183 ± 3.5956**	
Hypertension			0.3141
Yes	37	-7.5016 ± 5.3059	
No	94	-8.4078 ± 4.4520	
Diabetes			0.8050
Yes	15	-8.6199 ± 5.3399	
No	116	-8.0914 ± 4.6398	
Tumor subsite			0.6989
Supraglottic	52	-8.1244 ± 4.2122	
Glottic & Subglottic	79	-8.1701 ± 5.0302	
**pT stage**			**0.0029**

**T1 - T2**	**54**	**-9.4000 ± 5.4448**	
**T3 - T4**	**77**	**-7.2767 ± 3.9141**	
pN stage			0.3283
N0	90	-8.4682 ± 4.7903	
N+	41	-7.4577 ± 4.4933	
**pTNM stage***			**0.0167**
**I - II**	**41**	**-9.7544 ± 5.0619**	
**III - IV**	**90**	**-7.4220 ± 4.3711**	
Pathological differentiation			0.1051
Moderate & Poor	91	-8.5776 ± 4.8884	
Well	40	-7.1835 ± 4.1569	
**Tumor diameter**			**0.0168**
**> 3cm**	**58**	**-7.0433 ± 4.4707**	
**≤ 3cm**	**73**	**-9.0328 ± 4.7308**	

*According to the 8th American Joint Committee on cancer (AJCC) stage system.

Table S2. Multiple linear regression method with *F. nucleatum* content (-ΔCt) as the dependent variable, related to Figure 1.

Linear regression	*B*	*P* value	95% CI
**Drinking index**	**0.4053**	**< 0.0001**	**0.0004 - 0.0009**
**pTNM stage***	**0.2752**	**0.0011**	**0.5662 - 2.2058**
Age	0.1093	0.1859	-0.0320 - 0.1631
Hypertension	0.0285	0.7291	-1.398 - 1.9926
Smoking index	-0.0166	0.8374	-0.0009 - 0.0007
Sex	-0.0126	0.8795	-5.3130 - 4.5556
Diabetes	0.0103	0.9003	-2.2428 - 2.5466

Drinking index: the average gram of alcohol intaked per day × years spent drinking; Smoking index: the number of cigarettes smoked per day × years spent smoking. *According to the 8th American Joint Committee on cancer (AJCC) stage system.

Table S3. The correlation of *F. nucleatum* content (-ΔCt) and clinicopathologic factors in Cohort 2, related to Figure 1.

Characteristics	Total, n	*F. nucleatum* amount (-ΔCt)		*P*
		Low, n, %	High, n, %	
All cases, n	40	20 (50.00%)	20 (50.00%)	
Age, years				0.3332
≥ 60	24	14 (70.00%)	10 (50.00%)	
< 60	16	6 (30.00%)	10 (50.00%)	
Smoking history				> 0.9999
Yes	27	13 (65.00%)	14 (70.00%)	
No	13	7 (35.00%)	6 (30.00%)	
**Drinking history**				**0.0225**
**Yes**	**23**	**8 (40.00%)**	**16 (80.00%)**	
**No**	**17**	**12 (60.00%)**	**4 (20.00%)**	
Hypertension				> 0.9999
Yes	15	8 (40.00%)	7 (35.00%)	
No	25	12 (60.00%)	13 (65.00%)	
Diabetes				0.4872
Yes	2	2 (10.00%)	0 (0.00%)	
No	38	18 (90.00%)	20 (100.00%)	
Tumor subsite				> 0.9999
Supraglottic	16	8 (40.00%)	8 (40.00%)	
Glottic	24	12 (60.00%)	12 (60.00%)	
pT stage				0.7512
T1 - T2	18	10 (50.00%)	8 (40.00%)	
T3 - T4	22	10 (50.00%)	12 (60.00%)	

pN stage				> 0.9999
N0	23	11 (55.00%)	12 (60.00%)	
N+	17	9 (45.00%)	8 (40.00%)	
pTNM stage*				0.4801
I - II	11	7 (35.00%)	4 (20.00%)	
III - IV	29	13 (65.00%)	16 (80.00%)	
Pathological differentiation				> 0.9999
Well	6	3 (15.00%)	3 (15.00%)	
Moderate	34	17 (85.00%)	17 (85.00%)	
Tumor diameter				> 0.9999
> 3cm	13	7 (35.00%)	6 (30.00%)	
≤ 3cm	27	13 (65.00%)	14 (70.00%)	
Recurrence				**0.0011**
Yes	17	**3 (15.00%)**	**14 (70.00%)**	
No	23	**17 (85.00%)**	**6 (30.00%)**	
Cancer-specific death				
Yes	11	**2 (10.00%)**	**9 (45.00%)**	**0.0310**
No	29	**18 (90.00%)**	**11 (55.00%)**	

*According to the 8th American Joint Committee on cancer (AJCC) stage system.

Table S4. The correlation of *F. nucleatum* content (-ΔCt) and clinicopathologic factors in Cohort 3, related to Figure 6.

Characteristics	Total, n	*F. nucleatum* amount (-ΔCt)		*P*
		Low, n, %	High, n, %	
All cases, n	74	41 (55.41%)	33 (44.59%)	
Age, years				0.2436
≥ 60	41	20 (48.78%)	21 (63.64%)	
< 60	33	21 (51.22%)	12 (36.36%)	
Sex				0.5826
Male	71	40 (97.56%)	31 (93.94%)	
Female	3	1 (2.44%)	2 (6.06%)	
Smoking history				0.6301
Yes	46	24 (58.54%)	22 (66.67%)	
No	28	17 (41.46%)	11 (33.33%)	
**Drinking history**				**0.0182**
**Yes**	**42**	**18 (43.90%)**	**24 (72.73%)**	
**No**	**32**	**23 (56.10%)**	**9 (27.27%)**	
Hypertension				0.6301
Yes	28	17 (41.46%)	11 (33.33%)	
No	46	24 (58.43)	22 (66.67%)	
Diabetes				0.7439
Yes	10	5 (12.20%)	5 (15.15%)	
No	64	36 (87.80%)	28 (84.85%)	
Tumor subsite				0.6194
Supraglottic	24	12 (29.27%)	12 (36.36%)	
Glottic	50	29 (70.73%)	21 (63.64%)	

**pT stage**				**0.0187**
**T1 - T2**	**37**	**26 (63.41%)**	**11 (33.33%)**	
**T3 - T4**	**37**	**15 (36.59%)**	**22 (66.67%)**	
pN stage				0.5635
N0	59	34 (82.93%)	25 (75.76%)	
N+	15	7 (17.07%)	8 (24.24%)	
**pTNM stage***				**0.0182**
**I - II**	**32**	**23 (56.10%)**	**9 (27.27%)**	
**III - IV**	**42**	**18 (43.90%)**	**24 (72.73%)**	
Pathological differentiation				0.7989
Well	21	11 (26.83%)	10 (30.30%)	
Moderate & Poor	53	30 (73.17%)	23 (69.70%)	
Tumor diameter				0.4597
> 3cm	25	12 (29.27%)	13 (39.39%)	
≤ 3cm	49	29 (70.73%)	20 (60.61%)	
**Recurrence**				**0.0009**
**Yes**	**33**	**11 (26.83%)**	**22 (66.67%)**	
**No**	**41**	**30 (73.17%)**	**11 (33.33%)**	
**Cancer-specific death**				**0.0255**
**Yes**	**25**	**9 (21.95%)**	**16 (48.48%)**	
**No**	**49**	**32 (78.05%)**	**17 (51.52%)**	

*According to the 8th American Joint Committee on cancer (AJCC) stage system.

Table S5. Primer sequences used for qRT-PCR and FISH, related to STAR Methods.

Preimers	Forward primer (5' to 3')	Reverse primer (5' to 3')	Propose
F. nucleatum	CAACCATTACTTTAACTC TACCATGTTCA	GTTGACTTTACAGAAGGAGAT TATGTAAAAATC	qPCR
FUS664	CTTGTAGTT CCGC(C/T)TACCTC	N/A	FISH
EUB338	GCTGCCTCCCGTAGGAG T	N/A	FISH
has-miR-155-5p	AACCCCTATCACGATTAG CATTAA	N/A	FISH
has-miR-205-5p	CAGACTCCGGTGGAATG AAGGA	N/A	FISH
has-miR-155-5p	CGCTTAATGCTAATCGTG ATAGGGGTT	N/A	qPCR
has-miR-205-5p	TCCTTCATTCCACCGGAG TCT	N/A	qPCR
PGT	ATCCCCAAAGCACCTGG TTT	AGAGGCCAAGATAGTCCTGGT AA	qPCR
U6	GTGCTCGCTTCGGCAGC ACAT	N/A	qPCR
GAPDH	TGTAGTTGAGGTCAATGA AGGG	ACATCGCTCAGACACCATG	qPCR
TGFBR2	GTAGCTCTGATGAGTGC AATGAC	CAGATATGGCAACTCCCAGTG	qPCR
TLR4	GGATGAGGACTGGGTAA GGAATGA	AGCGGCTCTGGATGAAGTGC	qPCR

MYD88	GCCGCCGGATGGTGGTG GTTGT	TTGGTGCAGGGGTTGGTGTA GTCG	qPCR
E-cadherin	ATTTTTCCCTCGACACCC GAT	TCCCAGGCGTAGACCAAGA	qPCR
N-cadherin	TGCGGTACAGTGTAACT GGG	GAAACCGGGCTATCTGCTCG	qPCR
Vimentin	AGTCCACTGAGTACCGG AGAC	CATTTCACGCATCTGGCGTTC	qPCR
ZEB-1	GATGATGAATGCGAGTC AGATGC	ACAGCAGTGTCTTGTTGTTGT	qPCR
ADH1A	AGTCATCCCACTCGCTAT TCC	GTCCCCTGAGGATTGCTTACA	qPCR
ADH1B	GCTGGGGAATTGAAGCC AACA	CAGCATGTGTATGTTCAGGGC AAG	qPCR
ADH1C	TGCTACTGACTGGACGC ACG	CAGCCACAAGTTTGGGGACA GAT	qPCR
ADH4	AGTTCGCATTCAGATCAT TGCT	CTGGCCCAATACTTTCCACAA	qPCR
ADH5	ATGGCGAACGAGGTTAT CAAG	CATGTCCCAAGATCACTGGAA AA	qPCR
ADH6	ACAGGCCAAGTCATCAG ATGC	CCACAACCTTTATGCGAACTT CC	qPCR
ADH7	ACATGAGGCAACTGGGA TTGT	CATTGCATTCTCTACATTGTG GC	qPCR
CYP2E1	GTGATGCACGGCTACAA GG	GGGTGGTCAGGGAAAACCG	qPCR
